# Subvacuum environment‐enhanced cell migration promotes wound healing without increasing hypertrophic scars caused by excessive cell proliferation

**DOI:** 10.1111/cpr.13493

**Published:** 2023-05-01

**Authors:** Jian Jin, Bo‐han Pan, Kang‐an Wang, Shao‐Shuo Yu, Guo‐sheng Wu, He Fang, Bang‐hui Zhu, Yu Chen, Liang‐liang Zhu, Yan Liu, Zhao‐fan Xia, Shi‐hui Zhu, Yu Sun

**Affiliations:** ^1^ Department of Polymer Science Fudan University Shanghai China; ^2^ Department of Burn Surgery, Changhai Hospital The Naval Medical University Shanghai China; ^3^ 903rd Hospital of PLA Hangzhou China; ^4^ Department of Burns and Plastic Surgery, Ruijing Hospital, School of Medicine Shanghai Jiao Tong University Shanghai China

## Abstract

Cell migration and proliferation are conducive to wound healing; however, regulating cell proliferation remains challenging, and excessive proliferation is an important cause of scar hyperplasia. Here, we aimed to explore how a subvacuum environment promotes wound epithelisation without affecting scar hyperplasia. Human immortalized keratinocyte cells and human skin fibroblasts were cultured under subvacuum conditions (1/10 atmospheric pressure), and changes in cell proliferation and migration, target protein content, calcium influx, and cytoskeleton and membrane fluidity were observed. Mechanical calcium (Ca^2+^) channel blockers were used to prevent Ca^2+^ influx for reverse validation. A rat wound model was used to elucidate the mechanism of the subvacuum dressing in promoting healing. The subvacuum environment was observed to promote cell migration without affecting cell proliferation; intracellular Ca^2+^ concentrations and PI3K, p‐PI3K, AKT1, p‐AKT 1 levels increased significantly. The cytoskeleton was depolymerized, pseudopodia were reduced or absent, and membrane fluidity increased. The use of Ca^2+^ channel blockers weakened or eliminated these changes. Animal experiments confirmed these phenomena and demonstrated that subvacuum dressings can effectively promote wound epithelisation. Our study demonstrates that the use of subvacuum dressings can enhance cell migration without affecting cell proliferation, promote wound healing, and decrease the probability of scar hyperplasia.

## Graphical abstract

A subvacuum dressing can create a subvacuum environment in the area of the wound, and then calcium influx due to the opening of the mechanical calcium channel. Calcium influx leads to cytoskeleton depolymerisation through AKT1/PI3K signalling pathway. After cytoskeleton disaggregation, cells have stronger migration ability, thus promoting wound healing.

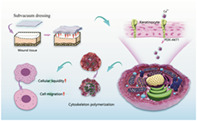

## 1 INTRODUCTION

Wounds are a common affliction in daily life. Acute wounds may be caused by burns and injuries, whereas the incidence of chronic wounds such as diabetic foot ulcers and pressure ulcers increases with aging.^1^, ^2^, ^3^ Thus, massive human and material resources are invested in the treatment of wounds. To promote wound healing, it is important to accelerate the proliferation and migration of local cells.^4^, ^5^, ^6^, ^7^ It also involves pathological and physiological activities such as vascularisation and inflammatory response.^8^, ^9^ However, cell proliferation is also associated with scar hyperplasia.^10^ Therefore, while the appropriate promotion of cell proliferation is conducive to wound healing, excessive proliferation may lead to pathological scarring. Unfortunately, existing technology cannot effectively regulate cell proliferation, which involves a balance between proliferation extent, order, time, and many other aspects; current measures can only increase the proliferation extent.^11^, ^12^, ^13^ Conversely, few studies have reported whether simple cell migration has a significant effect on scar hyperplasia. Cell migration plays several roles in wound healing; for example, the migration of keratinocytes is the basis of wound epithelisation, the migration of fibroblasts provides a scaffold for wound tissue growth, and the migration of vascular endothelial cells causes blood vessels to grow into endothelial cells.^14^, ^15^, ^16^, ^17^, ^18^, ^19^ Therefore, a novel method that can promote cell migration but have a minimal impact on cell proliferation is required.

Our team discovered during early research that the formation of an environment in which the pressure is below atmospheric pressure, also known as subvacuum pressure, in the local area of a wound will change the biological effect of wound cells, although this change is not linear. This article explored the changes of biological effects of wound cells in a subvacuum environment and explored the relevant underlying mechanisms.

## 2 MATERIALS AND METHODS

### 2.1 Reagents and materials

The subvacuum dressing used in this study was a dressing sponge (Shanghai Depeac Biotechnology Co., Ltd., Shanghai, China). Human immortalized keratinocyte (HaCat) and human fibroblast (HSF) cells were purchased from the Cell Bank of the Shanghai Institute for Biological Sciences (Shanghai, China). Alamar Blue was purchased from Thermo Fisher Scientific Inc. (Shanghai, China). PEX100 protein chips and kits were purchased from Full Moon BioSystems, Inc. (Sunnyvale, CA). Recombinant anti‐AKT1 (phospho S473) antibody (ab81283), anti‐PI3K gamma antibody (ab154598), anti‐PI3K (phospho Y607) (ab182651), anti‐tubulin antibody (ab61161), and anti‐vimentin antibody (ab92547) were purchased from Abcam Trading Co., Ltd. (Shanghai, China), while anti‐AKT1 (phospho Tyr326) (122661) was purchased from Heinupu Biotechnology Co., Ltd. (Shanghai, China). The Ca^2+^ detection kit Fluo 3‐Am (S1056) and 4′6‐diamidino‐2‐phenylindole (DAPI) (C1002) were purchased from Shanghai Beyotime Biotechnology Co., Ltd. (Shanghai, China). The anti‐fluorescence quenching sealing agent (0100‐01) was purchased from Southern Biotechnology Associates, Inc. (Birmingham, AL). Phalloidin (G1041) was purchased from Servicebio, Inc. (Wuhan, China). FITC, which was used to label goat anti‐rabbit IgG (BA1105), and Cy3 fluorescent dye, which was used to label goat anti‐mouse IgG (BA1031), were purchased from Wuhan BOSTER Biological Technology Co., Ltd. (Wuhan, China). TMA‐DPH (21492) was purchased from AAT Bioquest (Sunnyvale, CA). GdCl_3_ was purchased from Macklin (Shanghai, China).

#### 2.1.1 In vitro experiments

HaCat and HSF cell lines were used in this experiment. Cells in the subvacuum group were incubated in a cell culture incubator connected to an external pressure drainage system that provided a subvacuum environment through a water‐sealed bottle. The air pressure in the cell culture chamber was controlled by adjusting the water column height in the water‐sealed bottle. For all in vitro experiments, the pressures utilized in the cell culture incubator were 1/2, 1/5, 1/10, 1/15, and 1/20 atmospheres (atm). Cells in the control group were incubated with normal atmospheric pressure (1 atm). The barometric pressure used in the follow‐up study was comprehensively selected by comparing the effects on cell migration and proliferation.

#### 2.1.2 Cell migration experiment

A scratch test was performed to assess cell migration. Three fields in each cell well were randomly selected and imaged at 4, 12, 24, and 48 h after seeding. For each field, two lines were drawn to measure the cell‐to‐cell distance, and the mean values were calculated as the cell‐to‐cell spacing. The experiment was performed eight times. The mobility ratio was assessed using the following formula: (distance at 0 h − distance at measurement)/(distance at 0 h) × 100%. Cell mobility was then compared between the control group and the subvacuum group.

#### 2.1.3 Cell proliferation experiment

Cell proliferation was observed after staining with Alamar Blue. The absorbance of cells measured at 570 nm (A570) and 630 nm (A630) was measured every 2 days, and the degree of cell proliferation was calculated by dividing the A630 absorbance by the A570 absorbance. The experiment was performed eight times. The degree of cell proliferation was then compared between the control group and the subvacuum group.

EdU staining was used to verify cell proliferation. First, 50 μM EdU was added to each sample, followed by a 2‐h incubation period. Cells were then incubated at room temperature for 30 min in cell‐fixed solution (PBS containing 4% paraformaldehyde), followed by a 5‐min incubation period in 2 mg/mL glycine. Penetrant (0.5% PBS of Triton X‐100) was then added, and cells were incubated for 10 minutes. Cells were then incubated for 30 min in Apollo staining reaction solution. Penetrant was once again applied, followed by methanol for cleaning. Finally, cells were incubated for 30 min in Hoechst 33342 reaction solution and then washed with PBS. Under a fluorescence microscope, the number of positive cells in five random fields at ×400 magnification was counted and the average value was recorded.

### 2.2 Protein chip detection

HaCat and HSF cells were cultured under normal pressure and subvacuum conditions for 24 h. The sample protein was extracted and replaced with lysis/labelling buffer. The protein concentration was determined using a BCA kit, and the sample protein was labelled. The chip was sealed, incubated, and scanned at 532 nm with a power of 100%. Chip data were read and compared between groups after standardisation. A Kyoto Encyclopedia of Genes and Genomes (KEGG) pathway enrichment analysis was then performed. The chip scanner used was an Agilent SureScan DX Microarray Scanner with Genepix Pro 6.0 software (Axon Instruments, San Jose, CA).

### 2.3 Western blot analysis

The protein concentrations of PI3K, p‐PI3K, AKT1, and p‐AKT1 in HaCat and HSF cells were detected under subvacuum and normal pressure. Cells were cultured under subvacuum and normal pressure for 4, 12, 24, and 48 h. Cell sample proteins were then extracted, and 15 μg samples were loaded into each well. A 1.5 mm glass plate was used, and 8% separation glue and 5% concentrated glue were prepared. Electrophoresis was carried out at a constant voltage of 80 V until the loading buffer entered the separation gel. Electrophoresis continued at a constant voltage of 120 V until the loading buffer reached the bottom of the gel. The other laboratory settings included providing a constant current of 200 mA, transferring to the membrane for 2 h, and blocking for 1 h. The primary antibody was incubated and diluted to 1:5000. The primary antibody was then decolorized overnight at 4°C on a shaker. The secondary antibody was diluted with phosphate‐buffered saline with Tween® 20 at a ratio of 1:20,000, then incubated. An appropriate amount of ECL luminous solution was added for exposure. The samples were photographed, and ImageJ software (National Institutes of Health, Bethesda, MD) was used to read the grey values.

### 2.4 Detection of intracellular calcium concentration

HaCat and HSF cells were cultured for 4, 12, 24, and 48 h under subvacuum and normal pressure, and cell suspensions were prepared at 2 × 10^5^ cells/well overnight. Fluo 3‐Am was added to a final concentration of 10 μmol/L and incubated in the dark for 30 min. DAPI was used to stain the cell nuclei. The sealing process was performed using an anti‐fluorescence quenching sealing agent. The images were then observed under a fluorescence microscope, and the amount of fluorescence was quantified.

### 2.5 Cytoskeleton detection

HaCat and HSF cells were cultured for 4, 12, 24, and 48 h under subvacuum and normal pressure. The cells were grown on coverslips, and the aggregated microfilament cytoskeleton was stained with phalloidin (red). Specifically, the coverslips were fixed with 4% paraformaldehyde for 15 min, and phalloidin staining solution (5 μg/mL) was added. After incubation with DAPI in the dark for 5 min, the samples were stained and sealed with a sealing solution containing an anti‐fluorescence quenching agent. Images of the samples were observed under a fluorescence microscope and analysed quantitatively according to fluorescence intensity. Anti‐tubulin antibody (red) was used to label the aggregated tubulin, and anti‐vimentin antibody (green) was used to label the aggregated intermediate filaments (represented by vimentin). The coverslips were fixed with 4% paraformaldehyde, sealed with serum, and incubated with the primary antibody. Anti‐tubulin and anti‐vimentin antibodies were diluted at ratios of 1:100 and 1:200, respectively. The second fluorescent antibody was diluted to 1:100. Serum blocking and DAPI staining were performed. The images were observed under a fluorescence microscope and analysed quantitatively according to fluorescence intensity.

### 2.6 Membrane fluidity

HaCat and HSF cells were cultured for 4, 12, 24, and 48 h under subvacuum and normal pressure conditions. Then, 100 μL of 5 μM TMA‐DPH fluorescent probe solution was added and incubated for 10 min. The microplate reader was used for detection, with the following settings: EX = 355 nm, EM = 405. The smaller the fluorescence value, the greater the membrane fluidity.

### 2.7 Determining the concentration of mechanical Ca^2+^ channel blocker

HaCat and HSF cells were divided into the following treatment groups: (a) control; (b) GdCl_3_ 0.001 mM; (c) GdCl_3_ 0.01 mM; (d) GdCl_3_ 0.1 mM; (e) GdCl_3_ 1 mM; and (f) GdCl_3_ 10 mM. The treatment times were 12, 24, and 48 h. After the cells were cultured for the required time, 10 μL of Cell Counting Kit‐8 (CCK‐8) solution was added to each well and the plates were incubated at 37°C for 4 h. The optical density at 450 nm was measured for each well using a microplate reader, and the cell survival rate was calculated thrice. The highest concentration of GdCl_3_ that had no significant effect on cell survival was used as the concentration to block mechanical calcium (Ca^2+^) channels in subsequent experiments.

### 2.8 Reverse validation after using mechanical Ca^2+^ channel blockers

According to the above experiments, the concentration of the mechanical Ca^2+^ channel blocker GdCl_3_ was determined. Cells were cultured in the blockers under normal pressure and subvacuum conditions, and changes in the above indicators were observed.

### 2.9 Animal experiments

#### 2.9.1 Animals

We selected 60 clean‐grade male Sprague–Dawley rats (provided by the Experimental Centre of the Second Military Medical University; licence number, SCXK [Shanghai] 2012‐0003) aged 8–10 weeks and weighing 180–220 g. The rats were randomly divided into two groups, that is, the subvacuum dressing and control groups, with 30 rats in each group.

#### 2.9.2 Wound preparation

A circular full‐thickness skin defect model with an outer diameter of 3.0 cm and an inner diameter of 0.5 cm was prepared on the backs of the rats. The crawling‐style growth of skin islands was simulated using residual circular areas of skin in the middle of the wound. In addition, an anti‐contraction ring was used to fix the edge of the wound to prevent wound contraction and healing. The subvacuum dressing was used for dressing changes in the subvacuum group, whereas the control group used a dressing of the same material that was unable to produce a subvacuum environment. The dressings were changed every 3 days. At 1 and 2 weeks, 10 rats were then randomly selected, euthanized, and sampled. The crawling‐style growth of the central skin island in the circular wound was observed every time the dressing was changed. In addition, wound healing rates were recorded. The wound healing rate was calculated as follows: wound healing rate (%) = (initial wound area − wound area at dressing change)/initial wound area × 100.

#### 2.9.3 Immunofluorescence

Microtubules and intermediate filaments were analysed using immunofluorescence in the aforementioned tissue samples. The same antibodies were used, and corresponding tissue sections were prepared. Diluted primary and secondary antibodies were added to glass slides, incubated in a humidifying box at 20°C for 1 h, covered with aluminium foil, and kept away from light for 30 min to condense Gelvatol. Images of the samples were then observed under a fluorescence microscope and analysed quantitatively according to fluorescence intensity.

#### 2.9.4 Western blot analysis

The protein concentrations of PI3K, p‐PI3K, AKT1, and p‐AKT1 in wound tissue were detected by western blot analysis, as detailed in Section 2.3.

### 2.10 Statistical analyses

Data were expressed as the mean ± standard deviation. All data were statistically analysed with SPSS software (version 21.0; IBM Corp., Armonk, NY) using the paired samples Student's *t*‐test if the data were normally distributed; otherwise, the Wilcoxon signed rank sum test of paired samples was used. *P* < 0.05 indicated a statistically significant difference.

## 3 RESULTS

### 3.1 In vitro experiments

Cell proliferation and migration ability changed between subvacuum environments. At 1/2, 1/5, and 1/10 atm, cells only demonstrated an increase in migration ability, and the degree of increase was inversely proportional to the atmospheric pressure (Figure S1A,B,D,E). At 1/15 and 1/20 atm, cell proliferation ability increased, but migration ability did not increase significantly (Figure S1C,F). Therefore, the sub‐vacuum environment of 1/10 atm was further explored in this study.

Treatment of HaCat and HSF cells with ≤0.1 mM of the Ca^2+^ blocker GdCl_3_ for 48 h had no significant effect on the cell survival rate (*P* > 0.05); however, when the concentration was >0.1 mM, the blocker had a significant effect on the cell survival rate (*P* < 0.05; Figure S2). Hence, the highest concentration of the blocker that had no significant effect on cell survival rate was determined as 0.1 mM.

### 3.2 Cell migration and proliferation

**FIGURE 1:**
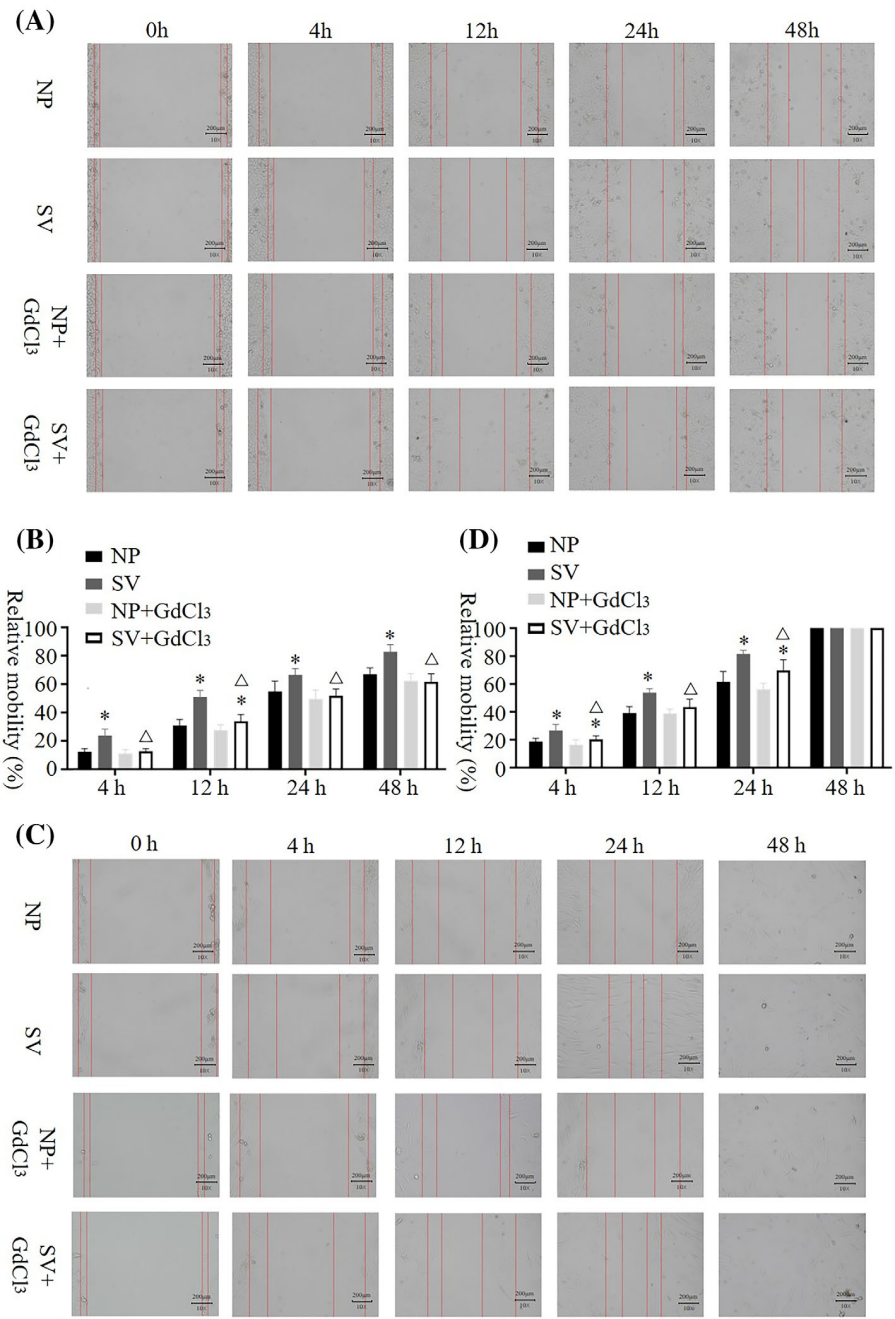
Migration ability of HaCat and HSF cells under normal pressure and in subvacuum environments. (A, B) Scratch test showing that subvacuum conditions promoted HaCat cell migration in the absence of the Ca^2+^ channel blocker GdCl_3_; the cell migration ratio at each time period was significantly higher than that under normal pressure (*n* = 8; *P* < 0.05). The addition of a blocker did not affect the migration ability of cells cultured under normal pressure (*P* > 0.05); however, the ability of the subvacuum environment to enhance cell migration was significantly weakened or eliminated (*P* < 0.05). (C, D) Scratch test showing that subvacuum conditions promoted HSF cell migration in the absence of the Ca^2+^ channel blocker GdCl_3_; the cell migration ratio at each time period was significantly higher than that under normal pressure (*n* = 8; *P* < 0.05). The addition of a blocker did not affect the migration ability of cells cultured under normal pressure (*P* > 0.05); however, the ability of the subvacuum environment to enhance cell migration was significantly weakened or eliminated (*P* < 0.05). * indicates a significant difference compared with the normal pressure without the blocker (*P* < 0.05). △ indicates a significant difference between the subvacuum environment with and without the blocker (*P* < 0.05). HaCat, human immortalized keratinocyte cells; HSF, human skin fibroblasts; NP, normal pressure; SV, subvacuum.

In the scratch test, when the Ca^2+^ channel blocker was not added, the mobility of HaCat and HSF cells was significantly higher when cultured in the subvacuum environment than when cultured under normal pressure (*P* < 0.05); however, the mobility of HSF cells reached 100% at 48 h. Under normal pressure, no significant difference was observed in the mobility of HaCat and HSF cells between the groups with and without blockers (*P* > 0.05). However, the migration rates of HaCat and HSF cells cultured in a subvacuum environment were significantly lower in the group with the blocker than in that without the blocker (*P* < 0.05). With the blocker, mobility was higher in the subvacuum environment than in the control group at 12 h for HaCat, and at 4 and 24 h for HSF cells (*P* < 0.05). No significant difference was observed at the other time periods (*P* > 0.05, Figure 1).

Finally, no significant differences in cell proliferation were observed between the subvacuum and control groups among HaCat cells on days 1 (*P* = 0.571), 3 (*P* = 0.198), 5 (*P* = 0.261), and 7 (*P* = 0.736), or among HSF cells (*P* = 0.543, *P* = 0.178, *P* = 0.679, *P* = 0.430, respectively; Table S1). EdU staining further confirmed that cell proliferation was not significantly affected (Figure S3).

### 3.3 Protein chip analysis

Images of the hybridized chips were obtained by scanning (Figure S4). After standardisation, the difference in phosphorus between groups was analysed, and the ratio between groups was determined to be 1.6 as the screening threshold. A total of 130 differential proteins of HSF cells and 153 differential proteins of HaCat cells were screened under subvacuum and normal pressure conditions (Tables S2 and S3). The differentially expressed proteins were mapped with the KEGG database pathways, and the probability of mapping the differential proteins to different pathways was calculated using the Fisher algorithm. The differential proteins of HSF and HaCat cells under micro‐negative and normal pressure were significantly enriched in the PI3K/AKT signalling pathway (Tables S4 and S5).

### 3.4 AKT1, p‐AKT, 1 PI3K, and p‐PI3K contents in cells

**FIGURE 2:**
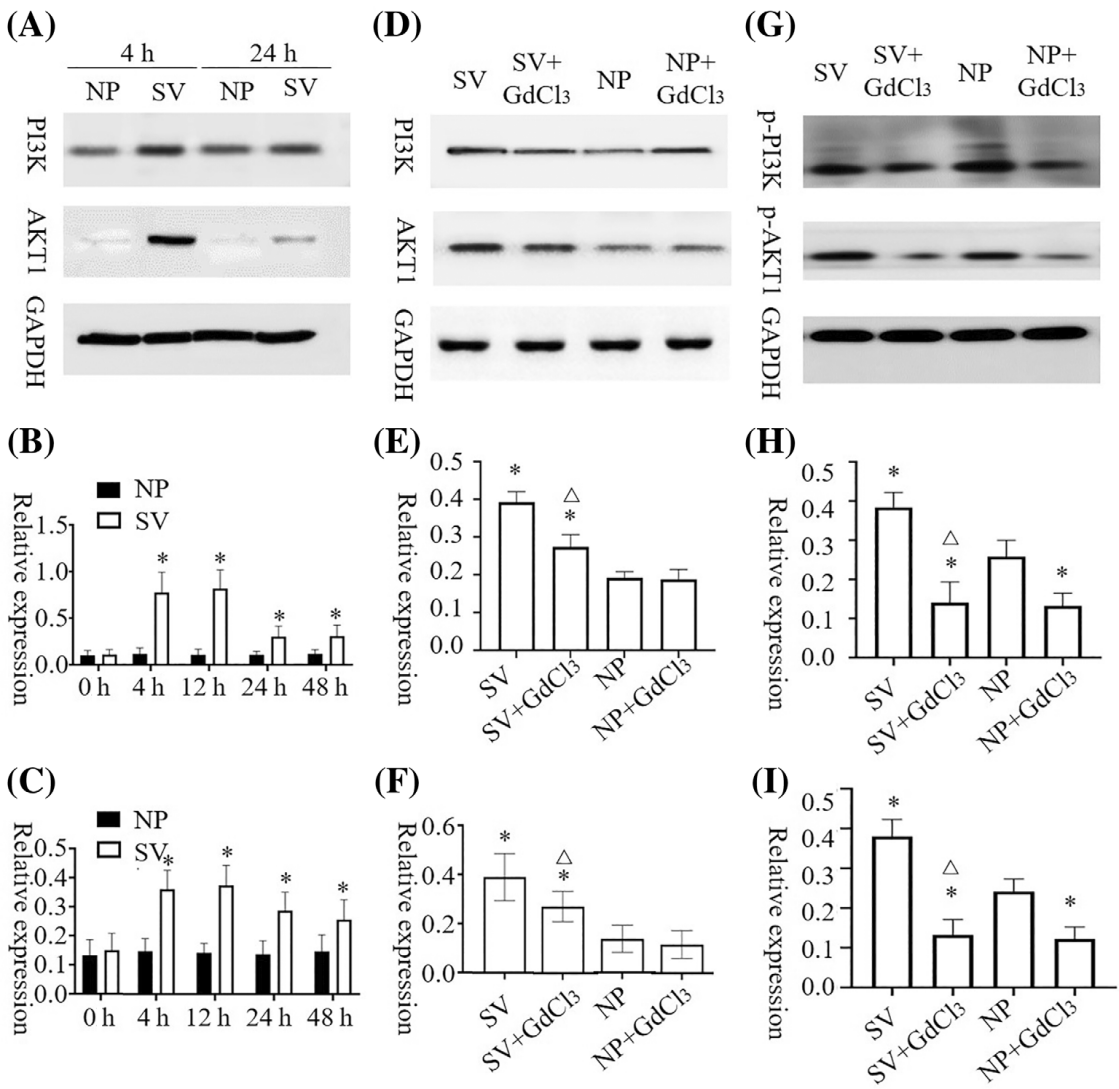
AKT1, p‐AKT1 and PI3K, p‐PI 3K expression changes in HaCat under normal pressure and subvacuum environments. (A–C) The expression of AKT1 and PI3K in HaCat increased significantly in the subvacuum environment (*P* < 0.05) then decreased after reaching a peak at 12–24 h, but to a higher level than that in the normal pressure environment. (D–F) The expression of AKT1 and PI3K in HaCat did not change significantly 4 h after adding the blocker (*P* > 0.05). Although the expression in the subvacuum environment was significantly higher than that in the normal pressure environment, it was still significantly lower than that without the blocker (*P* < 0.05). (G–I) The expression of p‐AKT1 and p‐PI3K in HaCat change significantly 4 h after adding the blocker (*P* < 0.05). After adding the blocker, the subvacuum did not significantly increase the p‐AKT1 concentration (*P* > 0.05), even lower than the normal pressure without blocker (*P* < 0.05). * indicates a significant difference compared with the normal pressure without the blocker (*P* < 0.05). △ indicates a significant difference between the subvacuum environment with and without the blocker (*P* < 0.05). HaCat, human immortalized keratinocyte cells; NP, normal pressure; SV, subvacuum.

**FIGURE 3:**
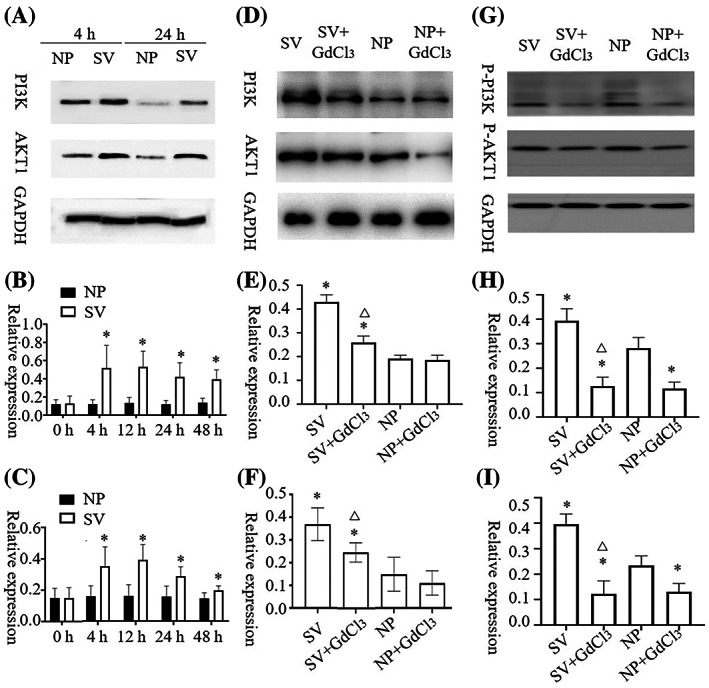
AKT1, p‐AKT1 and PI3K, p‐PI 3K expression changes in HSF under normal pressure and subvacuum environments. (A–C) The expression of AKT1 and PI3K in HSF increased significantly in the subvacuum environment (*P* < 0.05) then decreased after reaching a peak at 12–24 h, but to a higher level than that in the normal pressure environment. (D–F) The expression of AKT1 and PI3K in HSF did not change significantly 4 h after adding the blocker (*P* > 0.05). Although the expression in the subvacuum environment was significantly higher than that in the normal pressure environment, it was still significantly lower than that without the blocker (*P* < 0.05). (G–I) The expression of p‐AKT1 and p‐PI3K in HSF change significantly 4 h after adding the blocker (*P* < 0.05). After adding the blocker, the subvacuum did not significantly increase the p‐AKT1 concentration (*P* > 0.05), even lower than the normal pressure without blocker (*P* < 0.05). * indicates a significant difference compared with the normal pressure without the blocker (*P* < 0.05). △ indicates a significant difference between the subvacuum environment with and without the blocker (*P* < 0.05). HSF, human skin fibroblasts; NP, normal pressure; SV, subvacuum.

The AKT1 and PI3K contents of HaCat and HSF cells cultured under subvacuum conditions were significantly higher than those cultured under normal pressure (*P* < 0.05). AKT1 and PI3K contents peaked at 4–12 h and then remained stable before decreasing. AKT1 and PI3K contents were stable at 24 h but still significantly higher than those at normal pressure (Figures 2A–C and 3A–C, *P* < 0.05).

After the blocker was added, 4 h was selected as the time point for measuring AKT1, p‐AKT1, PI3K, and p‐PI3K contents. No significant difference in AKT1 concentrations was observed between HaCat cells with or without blockers under normal pressure (*P* > 0.05). However, GdCl_3_ treatment led to a significant decrease in p‐AKT1 content under normal pressure (*P*<0.05). After adding the blocker, the subvacuum environment did not significantly increase AKT1 or p‐AKT1 concentrations (*P* > 0.05), which were lower in the group with the blocker than in that without the blocker (*P* < 0.05). The change of PI3K and p‐PI3K concentrations in HaCat cells and AKT1, p‐AKT1, PI3K, and p‐PI3K concentrations in HSF cells were consistent with the change of AKT1 and p‐AKT1 concentrations in HaCat cells (Figures 2D–I and 3D–I).

### 3.5 Intracellular Ca^2+^ concentration

**FIGURE 4:**
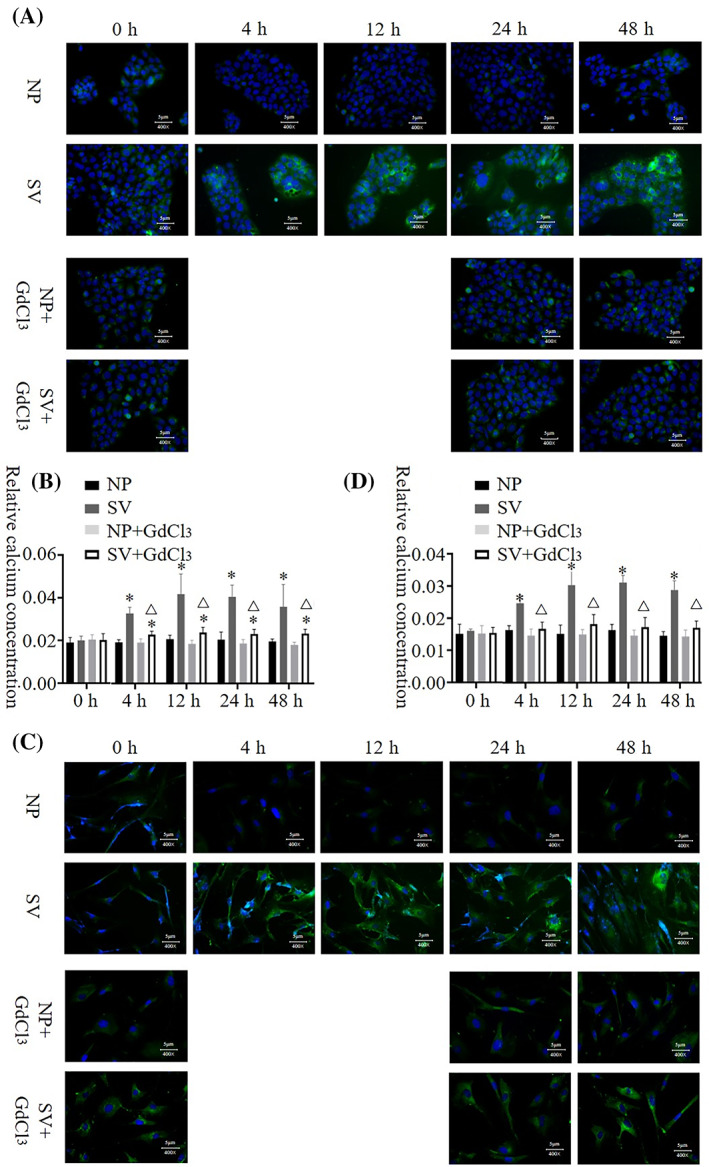
Intracellular Ca^2+^ concentration (green fluorescence) of HaCat and HSF cells under normal pressure and in subvacuum environments. (A, B) The intracellular Ca^2+^ concentration in HaCat cells increased significantly after 4 h under subvacuum conditions (*P* < 0.05). The addition of a Ca^2+^ channel blocker (GdCl_3_) had no significant effect on intracellular Ca^2+^ concentration under normal pressure (*P* > 0.05). Conversely, the ability of the subvacuum environment to promote an increase in intracellular Ca^2+^ concentration decreased significantly; however, it was still higher than that under normal pressure (*P* < 0.05). (C, D) The intracellular Ca^2+^ concentration in HSF cells increased significantly after 4 h under subvacuum conditions (*P* < 0.05). The addition of a blocker had no significant effect on intracellular Ca^2+^ concentration under normal pressure (*P* > 0.05). Conversely, the ability of the subvacuum environment to promote an increase in intracellular Ca^2+^ concentration decreased significantly; however, it was still higher than that under normal pressure (*P* < 0.05). * indicates a significant difference compared with the normal pressure environment without the blocker (*P* < 0.05). △ indicates a significant difference between the subvacuum environment with and without the blocker (*P* < 0.05). All immunofluorescence images were ×400 magnification. HaCat, human immortalized keratinocyte cells; HSF, human skin fibroblasts; NP, normal pressure; SV, subvacuum.

The Ca^2+^ concentration of HaCat and HSF cells at 4 h after being cultured was significantly higher in the subvacuum environment than under normal pressure (*P* < 0.05). In addition, the Ca^2+^ concentration in HaCat and HSF cells peaked at 24 h, then decreased. However, the concentration was still significantly higher than that under normal pressure (*P* < 0.05). After the blocker was added, the intracellular Ca^2+^ concentration in HaCat and HSF cells did not decrease significantly under normal pressure (*P* > 0.05). Conversely, under the subvacuum environment, the intracellular Ca^2+^ concentration in HaCat and HSF cells with the blocker was less than that in cells without the blocker. The Ca^2+^ concentration in HaCat cells was higher in the subvacuum environment than under normal pressure (*P* < 0.05), whereas no significant difference was observed in the Ca^2+^ concentration of HSF cells between the micro‐negative and normal pressure groups (*P* > 0.05; Figure 4).

### 3.6 Aggregation of the cytoskeleton

**FIGURE 5:**
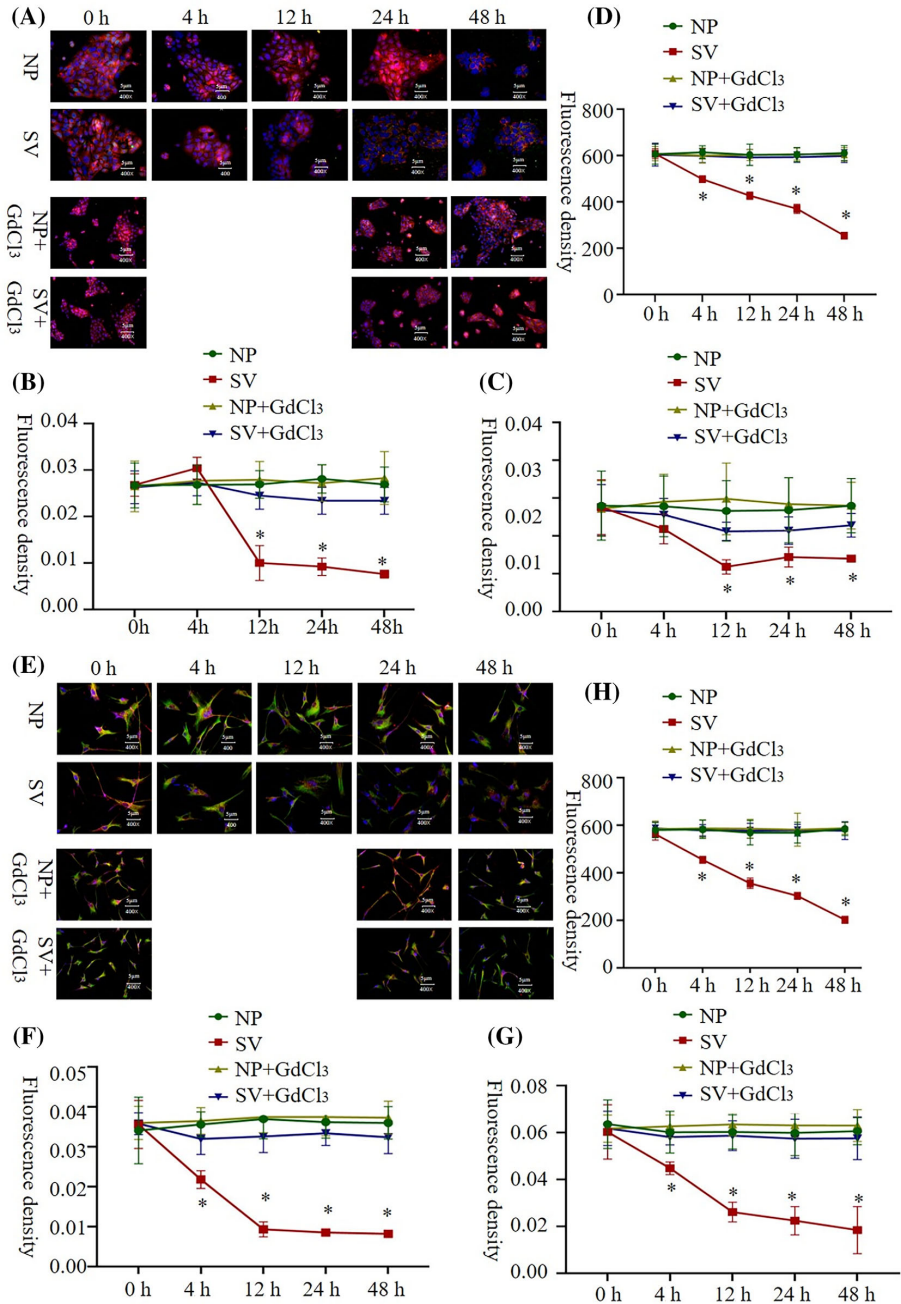
Changes in cytoskeleton (tubulin and intermediate filament) and membrane fluidity of HaCat cells under normal pressure and in subvacuum environments. (A–C) The aggregation of tubulin (red fluorescence staining) and intermediate filaments (green fluorescence) of HaCat cells was decreased due to depolymerisation (*P* < 0.05). In addition, the number of pseudopodia decreased, and the cytoskeletal structure became blurred. After adding the Ca^2+^ channel blocker GdCl_3_, the microfilament aggregation did not change significantly under normal pressure or subvacuum conditions (*P* > 0.05). (D) The fluidity of HaCat cell membranes began to increase significantly at 4 h under subvacuum conditions (*P* < 0.05). After adding the blocker, the subvacuum environment had no significant effect on the fluidity of the cell membrane (*P* > 0.05). (E–G) The aggregation of tubulin (red fluorescence staining) and intermediate filaments (green fluorescence) of HSF cells was similar to that of HaCat cells. (H) The fluidity of HSF cells was similar to that of HaCat cells. * indicates a significant difference compared with the normal pressure environment without the blocker (*P* < 0.05). All immunofluorescent images were acquired at ×400 magnification. HaCat, human immortalized keratinocyte cells; HSF, human skin fibroblasts; IF, intermediate filament; MF, microfilament; NP, normal pressure; SV, subvacuum.

In HaCat and HSF cells, the microfilament concentration indicated by phalloidin staining decreased in the subvacuum environment to a significantly lower value than that of the control group at 24 and 12 h (Figure S5, *P* < 0.05). The aggregation of microtubules and intermediate filaments observed in HaCat cells was similar to that of microfilaments. HSF cells demonstrated a similar aggregation pattern. In the subvacuum environment, the cytoskeletal components, including microfilament, microtubules, and intermediate filaments, all demonstrated a fuzzy structure with reduced or absent pseudopodia. After adding the Ca^2+^ channel blocker, the contents of microfilament, microtubules, and intermediate filaments in the cells were not significantly lower in the subvacuum environment than under normal pressure (*P* > 0.05). The cytoskeletal structure did not become fuzzy, and the pseudopodia were unchanged (Figure 5A–C,E–G).

### 3.7 Membrane fluidity

Fluidity increased continuously at 4 h (*P* < 0.05). After adding the Ca^2+^ channel blocker, the subvacuum environment did not lead to an increase in membrane fluidity, indicating no significant difference between the micro‐subvacuum group and normal pressure group (*P* > 0.05; Figure 5D,H).

### 3.8 Subvacuum dressing

**FIGURE 6:**
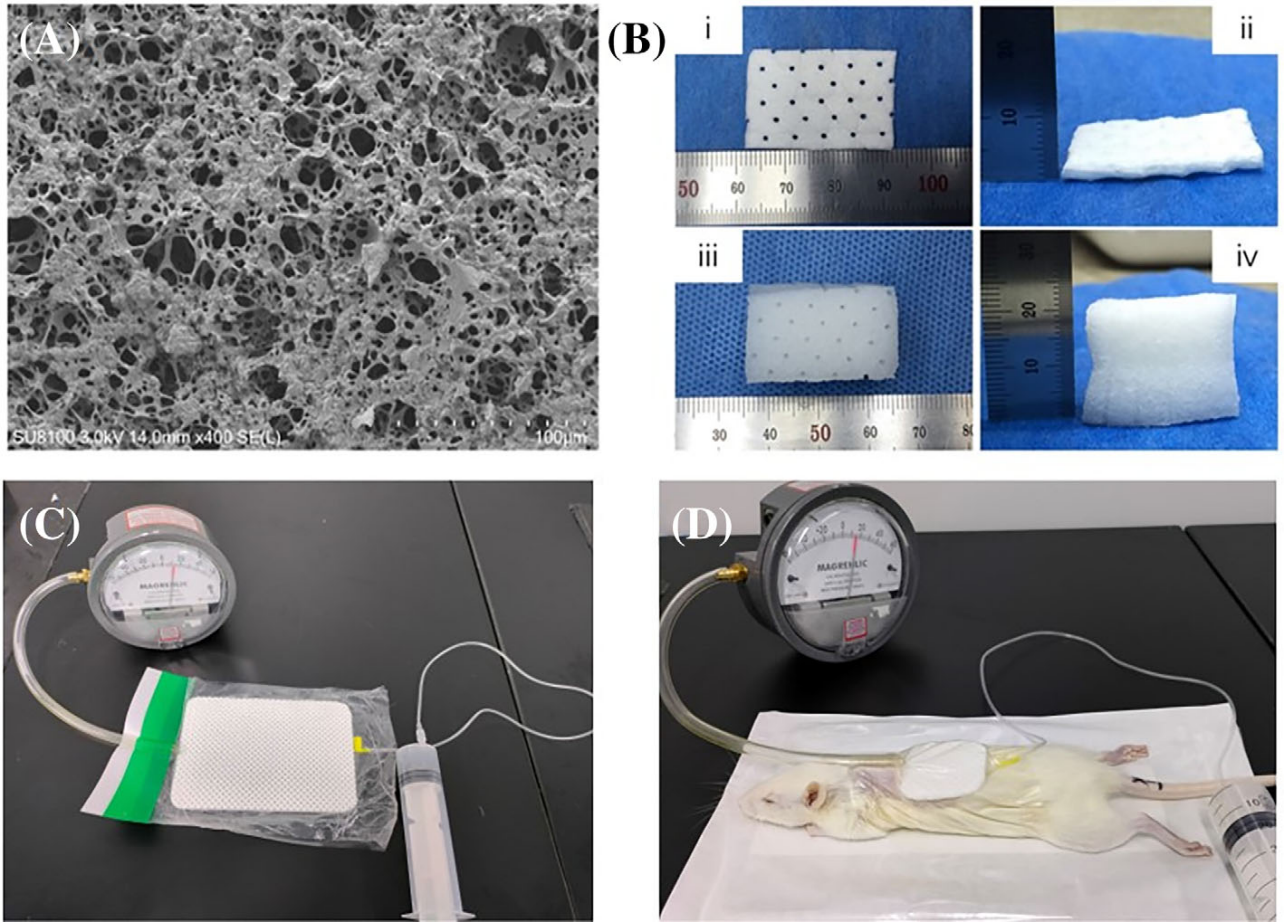
Characterisation of the subvacuum dressing. (A) Scanning electron microscope structure of the subvacuum dressing. (B) Compression and expansion of subvacuum dressing. (C,D) Subvacuum environment formed in vitro and in vivo.

Using polyvinyl alcohol as a raw material, a foam dressing with controllable porosity and pore size was prepared by high internal phase emulsions, which has a through‐hole structure and an opening degree of more than 90% (Figure 6A). The 2 cm thick dressing was compressed to 2 mm thick, and the wound was completely covered with a film during use to form a local closed environment (Figure 6B). The subvacuum dressing absorbed wound exudates, leading to the expansion of the dressing to its original thickness. This resulted in a 10‐fold increase of the space in the closed environment, thus forming a 1/10 atm subvacuum environment. In vivo and in vitro, the actual sub‐vacuum environment was found to be 0.1029 and 0.1047 atm, respectively, which are close to the theoretical value (Figure 6C,D).

### 3.9 Animal experiments

**FIGURE 7:**
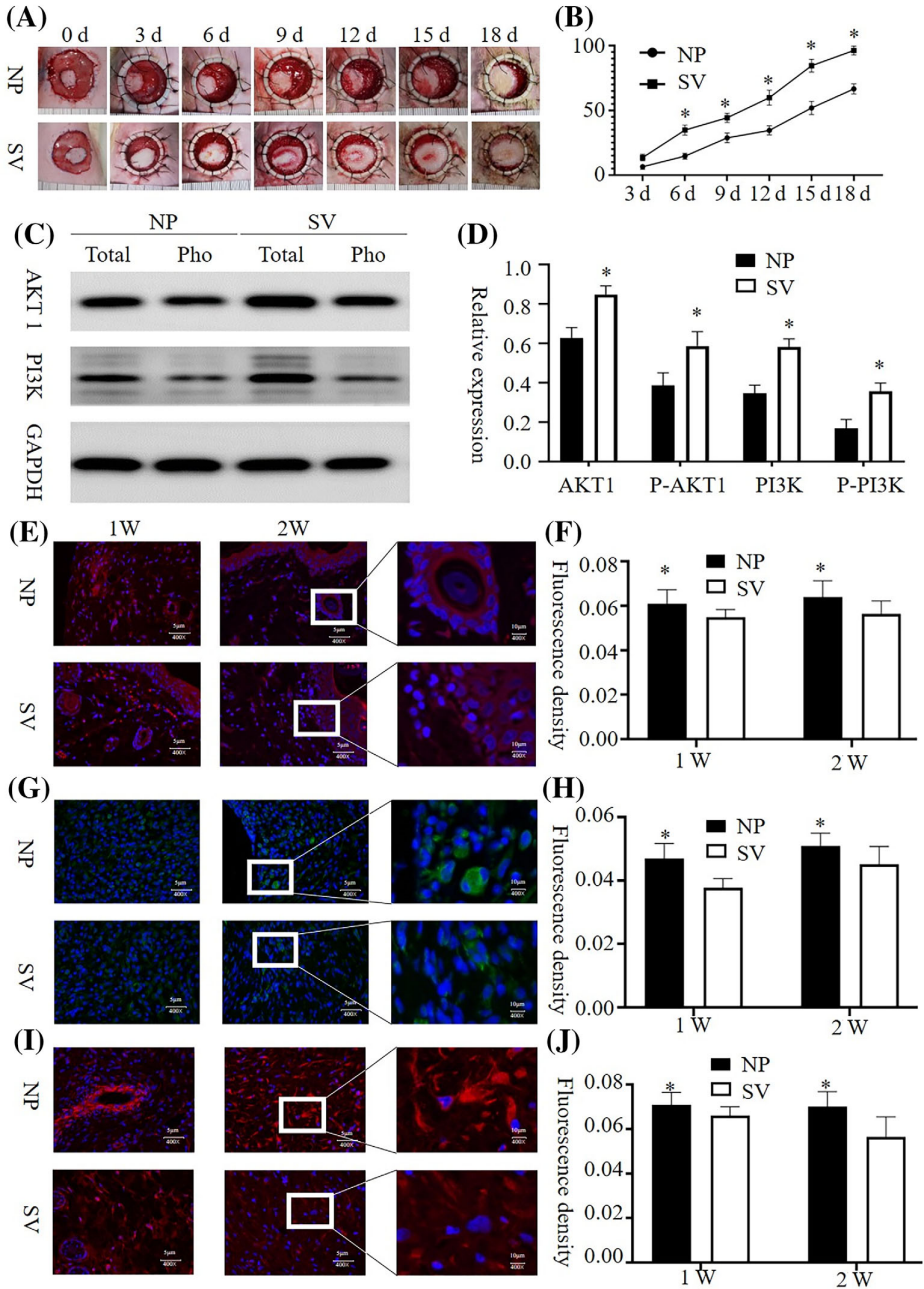
Effects of the subvacuum dressing compared with a similar dressing without the ability to generate a subvacuum. (A) Images of the wounds. The central skin island of all wounds expanded to all sides with crawling‐style growth to cover the wound, expanding significantly faster in the subvacuum group than the control group, healing completely on day 18. (B) Wound healing rate (%). The wound healing rate of the subvacuum group was significantly higher than that of the control group (*P* < 0.05). (C,D) The expression of AKT1, p‐AKT1, PI3K, and p‐PI3K in the wound tissue cells of the subvacuum group was significantly higher than that in the control group (*P* < 0.05). (E,F) The concentration of microfilaments (green fluorescence, white arrow) in the wound tissue of the subvacuum group was significantly lower than that of the control group (*P* < 0.05), and no pseudopodia were observed in the subvacuum group. (G,H) The concentration of tubulin (green fluorescence, white arrow) in wound tissue cells of the subvacuum group was significantly lower than that of the control group (*P* < 0.05), and no pseudopodia were observed in the subvacuum group. (I,J) The concentration of intermediate filaments (green fluorescence, white arrow) in wound tissue cells of the subvacuum group was significantly lower than that of the control group (*P* < 0.05), and no pseudopodia were observed in the subvacuum group. * indicates a significant difference compared with the control group (*P* < 0.05). HaCat, human immortalized keratinocyte cells; HSF, human skin fibroblasts; NP, normal pressure; SV, subvacuum.

Crawling‐style growth of skin islands across the wound was observed in both groups. The wound healing rate of the subvacuum group was higher than that of the control group (*P* < 0.05), however, the wound healing time of the subvacuum group was lower than that of the control group (*P* < 0.05). Immunofluorescence of wound tissues showed that AKT1 and PI3K concentrations in the subvacuum group were higher than those in the control group at 2 and 4 weeks, whereas the concentrations of aggregated microfilaments, microtubules, and intermediate filaments in the subvacuum group were significantly lower than those in the control group (*P* < 0.05). In addition, the cytoskeletal structure became fuzzy, and the number of pseudopodia decreased in the subvacuum group (Figure 7).

## 4 DISCUSSION

The application of various new materials in clinical practice has brought more and better options for the treatment of many diseases.^20^, ^21^, ^22^ Moisturizing dressings are widely used in clinics. Previous studies have found that when such dressings are compressed (often with the intention of enhancing their water absorption capacity), they produce better effects than ordinary moisturizing dressings, especially with regards to epithelialisation, after the wound is sealed with a film. Through analysis, it is believed that an environment below atmospheric pressure is formed during the expansion process after compression, which changes the biological effect of a cell and promotes wound healing. In response to this hypothesis, we decided to clarify that the change in the biological effect of the subvacuum environment on wound cells is more suitable to the needs of wound treatment through both in vitro cell experiments and in vivo animal experiments.

To provide a subvacuum environment for cells, a cell incubator was connected to a device that provided a continuous subvacuum. This device was patented in China (patent CN110229752A). Through the comparison of various subvacuum environments, the 1/10 atm sub‐vacuum environment was found to promote cell migration without affecting cell proliferation, which was the target of this study. This subvacuum atm value was also consistent with the average pressure produced by the subvacuum dressing used in this study. In the subvacuum environment, the cultured cells exhibited stronger mobility, which is crucial for wound healing. This is consistent with our hypothesis that the subvacuum dressing promotes epithelialisation by promoting the migration of wound cells. Thus, a subvacuum environment exhibits minor effects on the promotion of scar proliferation and will not increase the risk of scarring while accelerating the wound healing process, thereby improving the quality of healing. This finding is consistent with our previous clinical observations.

To further explore the mechanism by which the subvacuum environment promotes cell migration, protein chip technology was used to compare the changes in intracellular protein content under subvacuum and normal pressure environments. The results showed a significant increase in the content of proteins related to the PI3K/AKT1 signalling pathway, including AKT1, p‐AKT1 (phospho Tyr326), PI3K, and p‐PI3K (phospho Y607). This pathway is closely linked to cell migration and can promote cell migration by regulating the cytoskeleton.^23^, ^24^, ^25^, ^26^ In addition, Ca^2+^ is an important messenger in the activation of this signalling pathway.^27^, ^28^ Hence, our findings suggest that subvacuum dressings provide a subvacuum environment for wounds, which is conducive to Ca^2+^ influx, activation of the PI3K/AKT1 signalling pathway, and regulation of the cytoskeleton.^29^, ^30^ This study confirmed that the intracellular Ca^2+^ concentration increased significantly at 4 h under a subvacuum environment. Although the Ca^2+^ concentration displayed a decreasing trend after reaching a peak at 24 h, it still remained above a normal level. In addition, PI3K and AKT1 concentrations increased significantly. The increase in AKT1, p‐AKT1, PI3K, and p‐PI3K expression occurred more obvious than the significant change in Ca^2+^ concentration. Although no statistically significant change was observed, the early increase in Ca^2+^ concentration was strong enough to activate the downstream PI3K/AKT1 signalling pathway and amplify activation through the cascade reaction, which could result in an earlier change in AKT1, p‐AKT1, PI3K, and p‐PI3K concentrations. Nevertheless, it is possible that Ca^2+^ was not the only upstream signal of this pathway activated by the subvacuum environment; however, this hypothesis requires further evidence. With activation of the PI3K/AKT1 pathway, aggregation of the cytoskeleton increased in the early stage, which may be related to the increased synthesis of microfilaments, microtubules, and intermediate filaments. However, with further activation of the PI3K/AKT1 signalling pathway, the cytoskeleton depolymerized, and the pseudopodia decreased or disappeared at 4 h. Subsequently, the cytoskeleton remained in the depolymerized state. Hence, after 4 h, a significant increase was observed in the fluidity of the cell membrane, which is beneficial for promoting cell migration and wound healing.

The mechanical Ca^2+^ channel blocker GdCl_3_ was used for reverse validation in this study. The blocker significantly inhibited the increase in Ca^2+^ concentration; AKT1, p‐AKT1, PI3K, and p‐PI3K expression; membrane fluidity; and cytoskeleton depolymerisation caused by the subvacuum environment. In some cases, no obvious difference was observed between the subvacuum group and normal pressure group. These findings suggest that subvacuum conditions could activate downstream signalling pathways by developing mechanical Ca^2+^ channels, thereby increasing intracellular Ca^2+^ concentration.

This study outlines the design for a unique wound model via animal experiments using an annular full‐thickness skin defect model. The outer ring was fixed with an anti‐contraction ring to avoid contraction healing, which could affect the experimental observations. The inner part was left with a skin island, which was conducive for observing the epithelialisation promoted by the crawling‐style growth of skin islands related to cell migration.^31^, ^32^ When dressing with the same material under microdynamic negative pressure (where the influence of moisture and other variables were removed), the wound healing time was significantly reduced, whereas the wound healing rate was significantly increased. In addition, the contents of AKT1, p‐AKT1, PI3K, p‐PI3K increased in the wound tissue, and the cytoskeleton was depolymerized, observations that are consistent with the results of the cell experiments. These findings suggest that the subvacuum dressing is more conducive to wound healing than ordinary moisturizing dressings due to the subvacuum environment generated by the subvacuum dressing.

## 5 CONCLUSION

**FIGURE 8:**
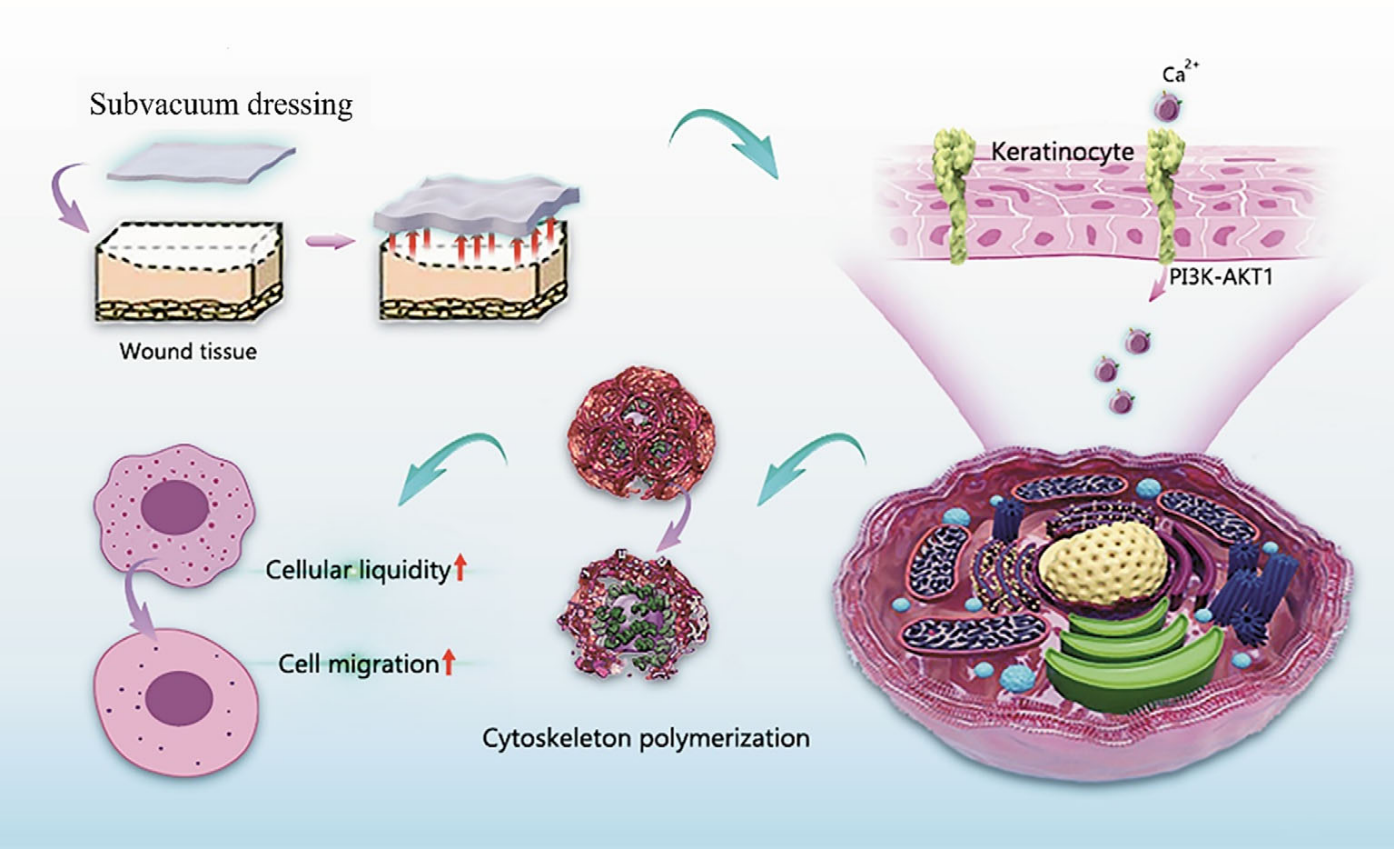
The underlying mechanism of the subvacuum‐mediated promotion of cell migration.

Through cell and animal experiments, this study showed that subvacuum dressing can promote wound healing by creating a subvacuum environment. Its underlying mechanism involves opening mechanosensitive Ca^2+^ channels by activating the PI3K/AKT1 signalling pathway, regulating the cytoskeleton, increasing cell membrane fluidity, and promoting cell migration (Figure 8). Moreover, cell proliferation was not affected during these processes. Therefore, subvacuum dressings can promote wound healing without increasing the risk of scar hyperplasia.

## AUTHOR CONTRIBUTIONS

Conceptualisation: Shi‐hui Zhu, Yu Sun, Jian Jina, Yan Liu; Methodology: Jian Jina, Bo‐han Pan, Kang‐an Wang, Shao‐Shuo Yu, Guo‐sheng Wu, Yan Liu; Cell experiment: Jian Jina, Bo‐han Pan, He Fang, Bang‐hui Zhu; Animal experiment: Jian Jina, Kang‐an Wang, Bang‐hui Zhu, Yu Chen; Data collation and analysis: Jian Jina, Liang‐liang Zhu; Funding acquisition: Zhao‐fan Xia, Shi‐hui Zhu, Yu Sun, Jian JinaL; Project administration: Shi‐hui Zhu, Zhao‐fan Xia, Yan Liu; Supervision: Zhao‐fan Xia, Yan Liu, Liang‐liang Zhu; Writing of the original draft: Jian Jina, Liang‐liang Zhu, Yu Sun; Writing, reviewing & editing of the manuscript: Shi‐hui Zhu, Yu Sun, Yan Liu, Liang‐liang Zhu.

## FUNDING INFORMATION

We received funding from the following institutions: National Key R&D Program of China (2019YFA0110600, 2019YFA0110601, 2019YFA0110602, 2019YFA0110603); National Natural Science Foundation of China (81772125, 81930057, 81772076); CAMS Innovation Fund for Medical Sciences (2019‐I2M‐5‐076); Medical and health science and technology project of Hangzhou (B20200432); Hangzhou Science and Technology Plan Development Project (20210133X01). The funding bodies had no role in the design of the study; collection, analysis, and interpretation of data; and in writing the manuscript.

## CONFLICT OF INTEREST STATEMENT

The authors declare that they have no competing interests.

## Supporting information


**Data S1.** Supporting Information.

## Data Availability

The data that support the findings of this study are available from the corresponding author upon reasonable request.
