# Supplementary material for: Subvacuum environment‐enhanced cell migration promotes wound healing without increasing hypertrophic scars caused by excessive cell proliferation
Source: Cell Prolif. 2023 May 1;56(11):e13493. doi: 10.1111/cpr.13493 (PMC10623940; doi:10.1111/cpr.13493)
Supplement: Supplementary file 1 — Data S1. Supporting Information. [file CPR-56-e13493-s001.docx]

**SUPPLEMENTARY MATERIALS**


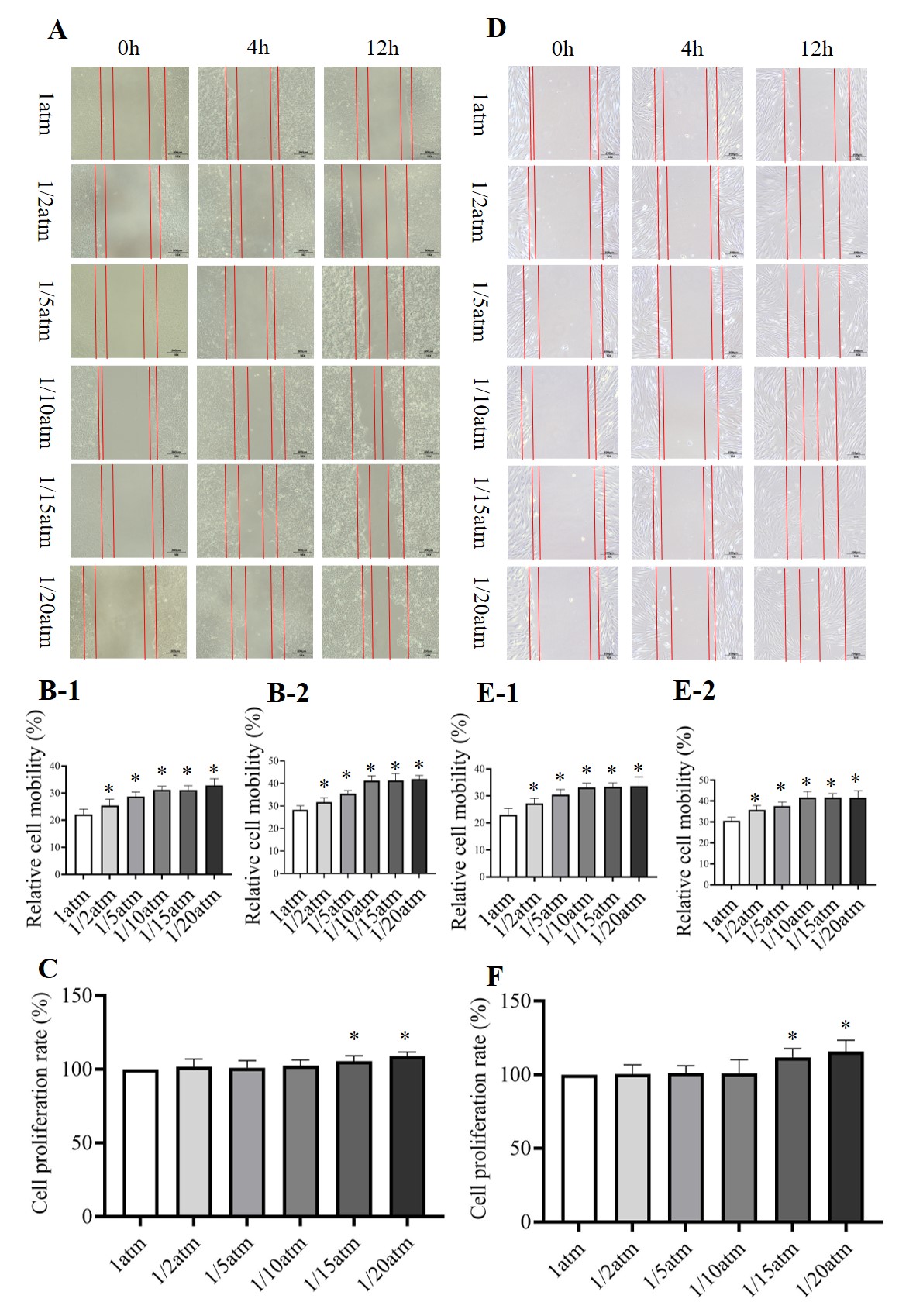


**Supplementary Figure S1.** **Migration ability and proliferation ability of HaCat and HSF cells under normal pressure and in subvacuum environments with different pressures. (A, B**) Scratch test showing that a subvacuum environment promoted HaCat cell migration; the cell migration ratio at each time period was significantly higher than that under normal pressure (n = 8; *P* < 0.05). B-1 for 4h, B-2 for 12h. (**C**) MTT test showing that 1/15 atm and 1/20 atm promoted HaCat cell proliferation (n = 8; *P* < 0.05); 1/2, 1/5, and 1/10 atm did not promote HaCat cell proliferation (n = 8; *P*＞0.05). **(D, E**) Scratch test showing that a subvacuum environment promoted HSF cell migration; the cell migration ratio at each time period was significantly higher than that under normal pressure (n = 8; *P* < 0.05). E-1 for 4h, E-2 for 12h. (**F**) MTT test showing that 1/15 atm and 1/20 atm promoted HSF cell proliferation (n = 8; *P* < 0.05); 1/2, 1/5, and 1/10 atm did not promote HSF cell proliferation (n = 8; *P*＞0.05). * indicates a significant difference compared with the normal pressure without the blocker (*P* < 0.05). atm, standard atmosphere; HaCat, human immortalized keratinocyte cells; HSF, human skin fibroblasts; MTT, 3-(4,5-dimethylthiazol-2-yl)-2,5-diphenyltetrazolium bromide


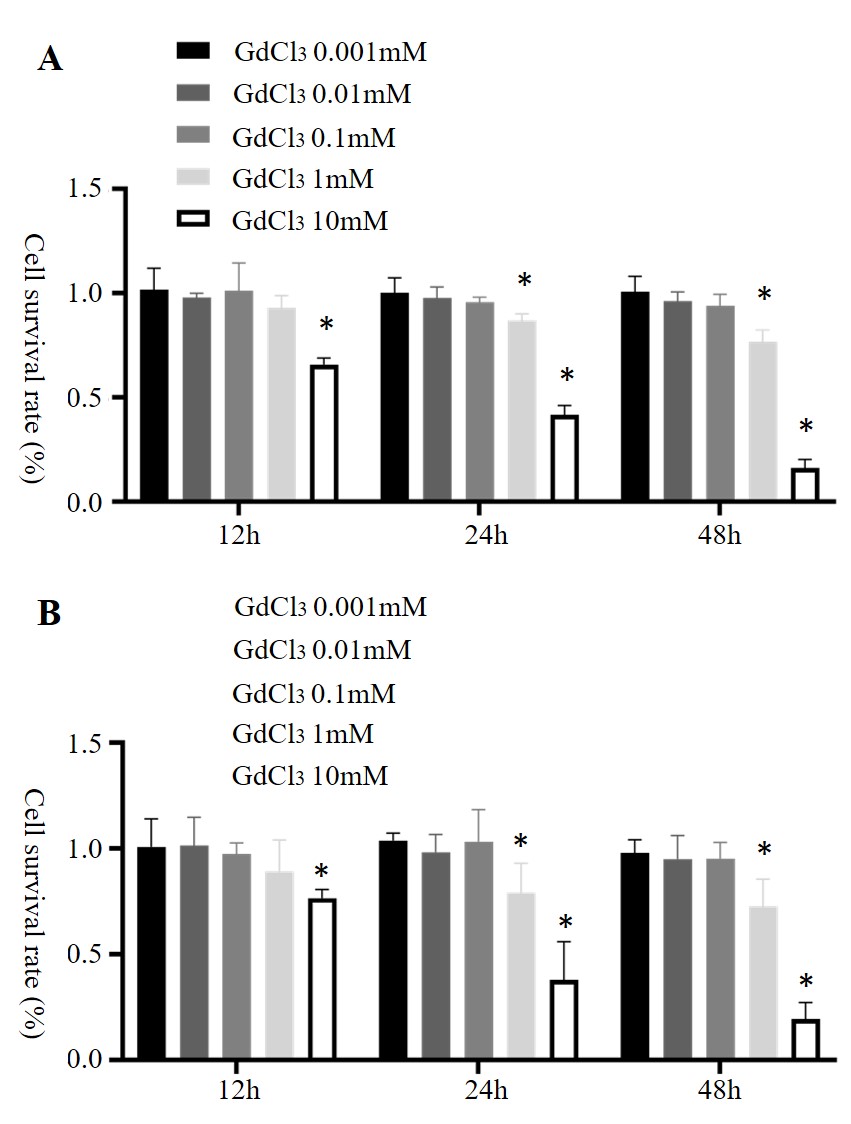


**Supplementary Figure S2.** **Effects of different GdCl_3_ concentrations on the survival rate of (A) HaCat and (B) HSF cells.** At a concentration of 0.1 mM, the Ca^2+^ channel blocker GdCl_3_ had no significant effect on the survival rate of HaCat or HSF cells (*P* > 0.05); however, increasing GdCl_3_ concentration significantly reduced the survival rate of HaCat and HSF cells (*P* < 0.05). * indicates a significant decrease in the cell survival rate (*P* < 0.05). HaCat, human immortalized keratinocyte cells; HSF, human skin fibroblasts.


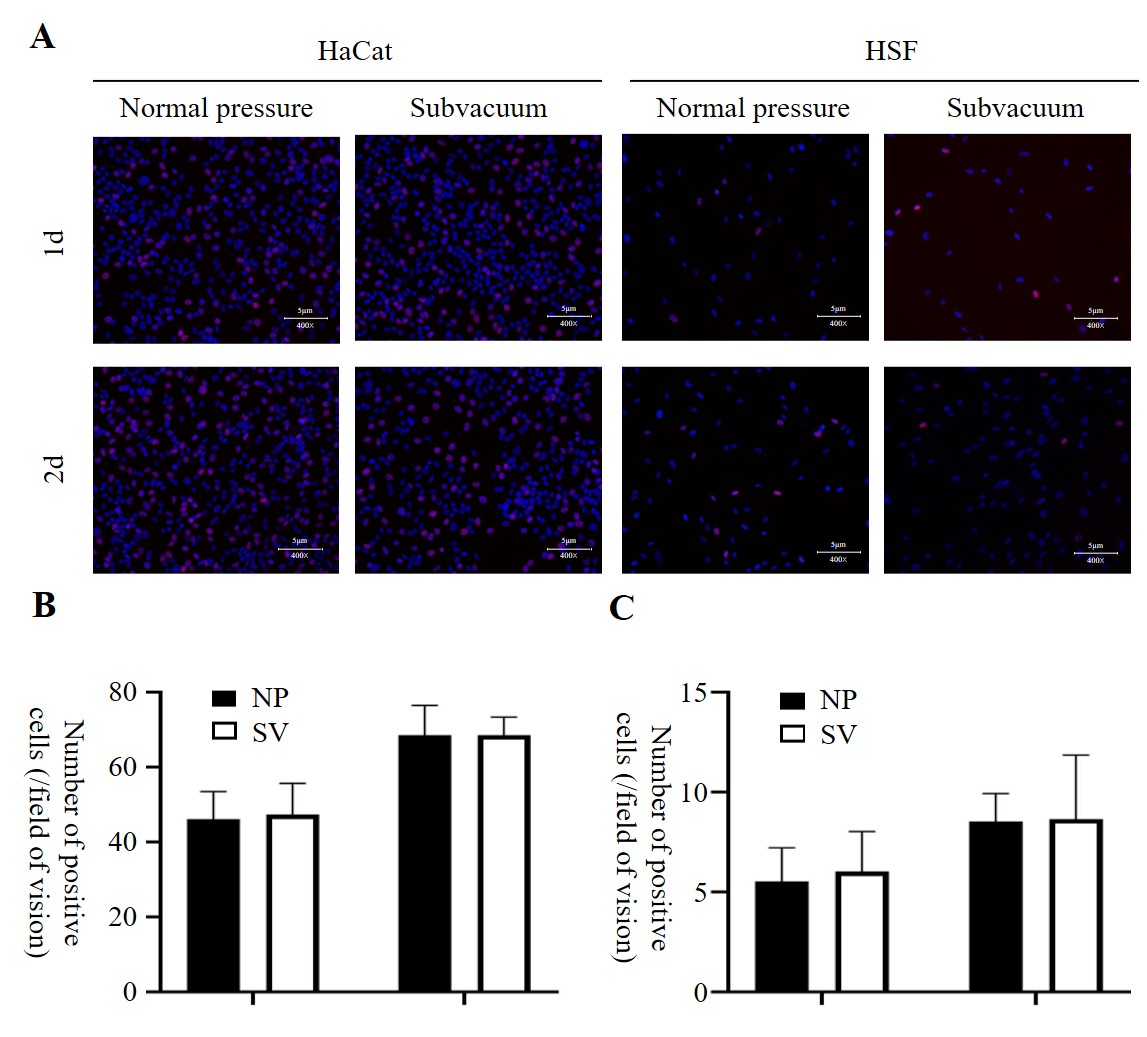


**Supplementary Figure S3.** **EdU staining was used to verify the effect of a subvacuum environment on cell proliferation.** (A) EdU fluorescence staining showed that a subvacuum environment had no significant effect on HaCat (B) and HSF (C) cells. NP, normal pressure; SV, Subvacuum; HaCat, human immortalized keratinocyte cells; HSF, human skin fibroblasts.


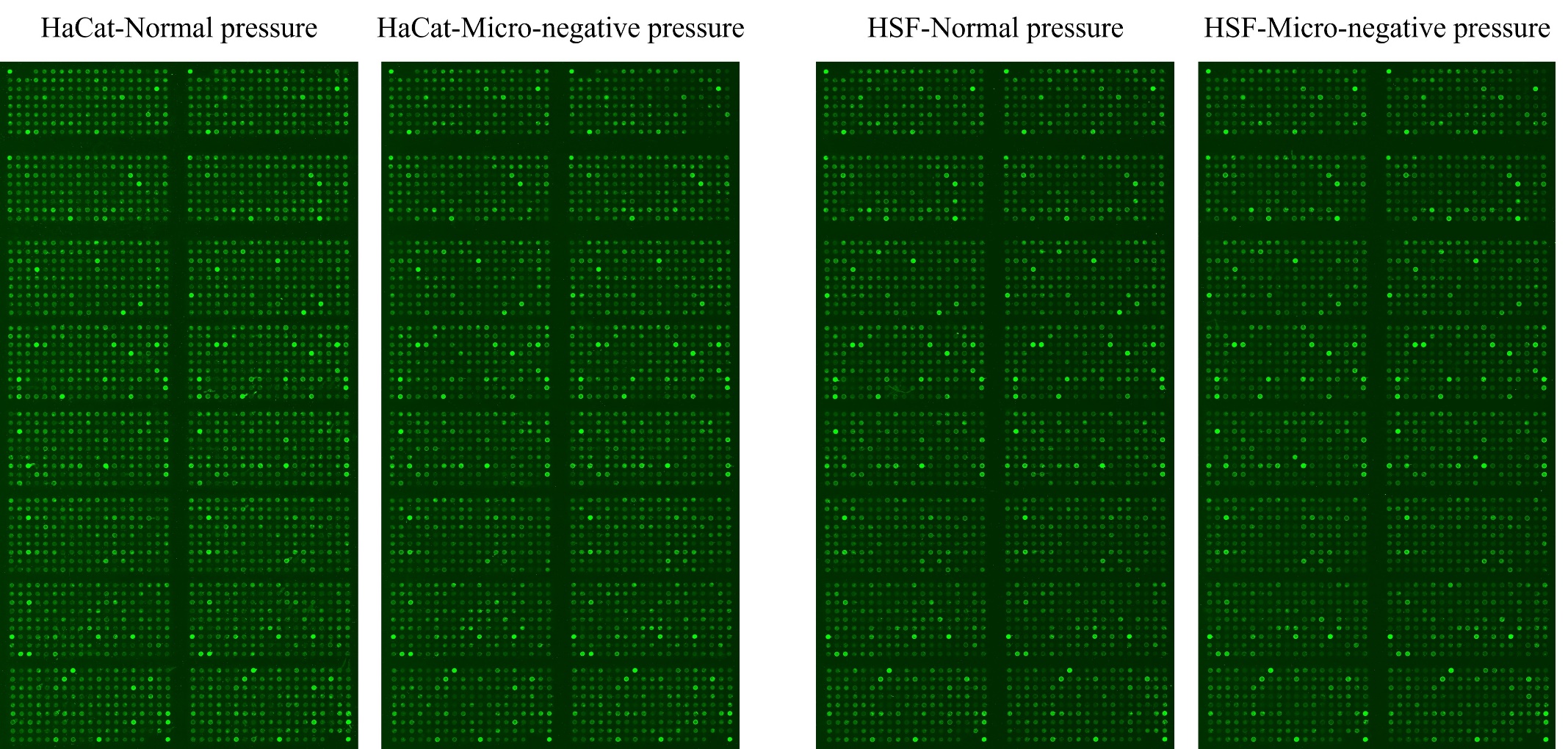


**Supplementary Figure S4.** **Protein chip results of HaCat and HSF cells under micro-negative pressure and normal pressure.**

HaCat, human immortalized keratinocyte cells; HSF, human skin fibroblasts.


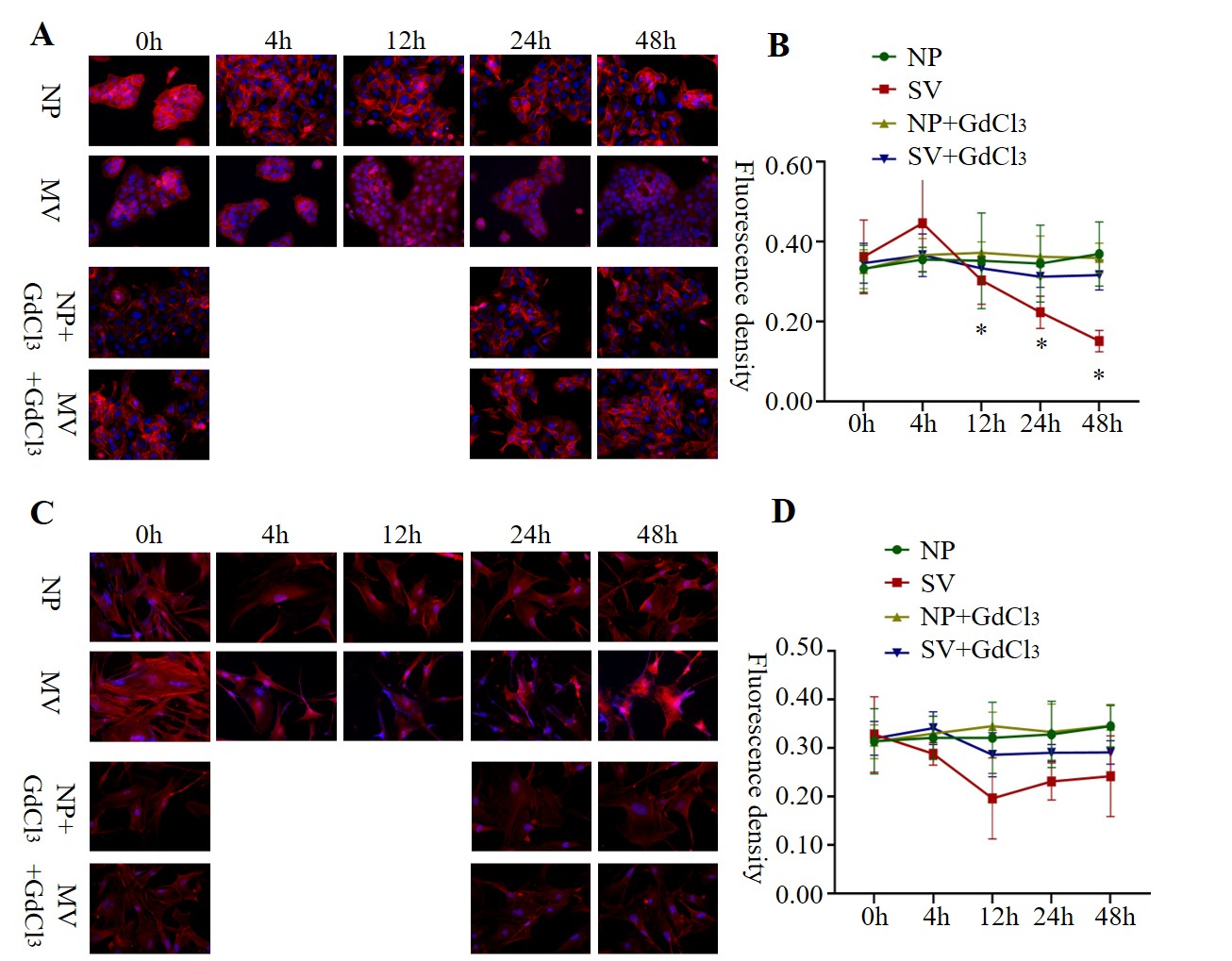


**Supplementary Figure S5.** **Microfilament changes in HaCat and HSF cells under normal pressure and in subvacuum environments. (A, B)** The concentration of microfilament in HaCat cells (red fluorescent staining of phalloidin) increased in the early stage under subvacuum conditions and then decreased due to depolymerization (*P* < 0.05). In addition, the number of pseudopodia decreased and the cytoskeletal structure became blurred. After adding the Ca^2+^ channel blocker GdCl_3_, the microfilament aggregation did not change significantly under normal pressure or subvacuum conditions (*P* > 0.05). **(C, D)** The concentration of microfilaments in HSF cells (red fluorescent staining of phalloidin) decreased due to depolymerization (*P* < 0.05). * indicates a significant difference compared with the normal pressure environment without the blocker (*P* < 0.05). All immunofluorescent images were acquired at 400 × magnification. NP, normal pressure; SV, Subvacuum; HaCat, human immortalized keratinocyte cells; HSF, human skin fibroblasts; IF, intermediate filament; MF, microfilament.

**Supplementary Table S1.** **Proliferation of human immortalized keratinocytes and human fibroblast cells**

Proliferation of human immortalized keratinocytes (X ± S, n = 8)

|  | **First day** | **Third day** | **Fifth day** | **Seventh day** |
| --- | --- | --- | --- | --- |
| **CG** | 1.92 ± 0.01 | 2.50 ± 0.05 | 4.55 ± 0.77 | 5.28 ± 0.78 |
| **NPG** | 1.89 ± 0.12 | 2.37 ± 0.27 | 4.91 ± 0.41 | 5.18 ± 0.28 |

Proliferation of human fibroblast cells (X ± S, n = 8)

|  | **First day** | **Third day** | **Fifth day** | **Seventh day** |
| --- | --- | --- | --- | --- |
| **CG** | 1.79 ± 0.04 | 1.97 ± 0.06 | 3.22 ± 0.22 | 4.14 ± 0.20 |
| **NPG** | 1.74 ± 0.22 | 1.94 ± 0.03 | 3.26 ± 0.15 | 4.06 ± 0.18 |

CG, control group; NPG, negative pressure group; S, standard deviation.

**Supplementary Table S2.** **Comparison of protein content in HSF under micro-negative pressure and normal pressure**

| **Name** | **Gene symbol** | **Uniprot** | **HSF**  **micro-negative pressure_Phos/ Unphos** | **HSF normal pressure_Phos/ Unphos** | **FC ≥ 1.6_HSF micro-negative _vs_HSF normal pressure** |
| --- | --- | --- | --- | --- | --- |
| CD19 (Phospho-Tyr531) | *CD19* | P15391 | 9.98 | 1.69 | 5.92 |
| Synuclein alpha (Phospho-Tyr133) | *SNCA* | P37840 | 2.82 | 0.76 | 3.69 |
| PDGFR alpha (Phospho-Tyr849) | *PDGFRA* | P16234 | 2.92 | 0.94 | 3.11 |
| RyR2 (Phospho-Ser2808) | *RYR2* | Q92736 | 0.94 | 0.33 | 2.84 |
| Connexin 43 (Phospho-Ser367) | *GJA1* | P17302 | 2.29 | 0.82 | 2.80 |
| Rel (Phospho-Ser503) | *REL* | Q04864 | 3.17 | 1.17 | 2.70 |
| Chk1 (Phospho-Ser317) | *CHEK1* | O14757 | 1.81 | 0.67 | 2.68 |
| EGFR (Phospho-Tyr1092) | *EGFR* | P00533 | 2.52 | 0.96 | 2.61 |
| CD3Z (Phospho-Tyr142) | *CD247* | P20963 | 0.87 | 0.34 | 2.58 |
| PAK2 (Phospho-Ser192) | *PAK2* | Q13177 | 3.10 | 1.24 | 2.50 |
| BAD (Phospho-Ser91/128) | *BAD* | Q92934 | 0.95 | 0.39 | 2.44 |
| Stathmin 1 (Phospho-Ser37) | *STMN1* | P16949 | 2.00 | 0.86 | 2.33 |
| p38 MAPK (Phospho-Tyr322) | *MAPK14* | Q16539 | 1.36 | 0.61 | 2.23 |
| HDAC8 (Phospho-Ser39) | *HDAC8* | Q9BY41 | 2.03 | 0.91 | 2.23 |
| PLC beta3 (Phospho-Ser1105) | *PLCB3* | Q01970 | 1.31 | 0.59 | 2.22 |
| BTK (Phospho-Tyr223) | *BTK* | Q06187 | 1.16 | 0.53 | 2.20 |
| IRS-1 (Phospho-Ser794) | *IRS1* | P35568 | 2.39 | 1.09 | 2.19 |
| Keratin 18 (Phospho-Ser33) | *KRT18* | P05783 | 1.94 | 0.89 | 2.19 |
| Myc (Phospho-Thr58) | *MYC* | P01106 | 2.26 | 1.04 | 2.18 |
| PTEN (Phospho-Ser370) | *PTEN* | P60484 | 0.84 | 0.40 | 2.13 |
| Cyclin B1 (Phospho-Ser126) | *CCNB1* | P14635 | 0.77 | 0.36 | 2.12 |
| IKK-alpha/beta (Phospho-Ser180/181) | *CHUK/IKBKB* | O15111/O14920 | 0.46 | 0.22 | 2.11 |
| MKK3/MAP2K3 (Phospho-Ser189) | *MAP2K3* | P46734 | 0.92 | 0.44 | 2.06 |
| IRS-1 (Phospho-Ser323) | *IRS1* | P35568 | 0.61 | 0.30 | 2.05 |
| Pyk2 (Phospho-Tyr881) | *PTK2B* | Q14289 | 1.05 | 0.52 | 2.00 |
| MITF (Phospho-Ser73) | *MITF* | O75030 | 1.07 | 0.56 | 1.93 |
| G3BP-1 (Phospho-Ser232) | *G3BP1* | Q13283 | 1.91 | 1.00 | 1.91 |
| Merlin (Phospho-Ser10) | *NF2* | P35240 | 3.15 | 1.67 | 1.89 |
| Synuclein alpha (Phospho-Tyr125) | *SNCA* | P37840 | 1.14 | 0.62 | 1.86 |
| p130Cas (Phospho-Tyr410) | *BCAR1* | P56945 | 0.97 | 0.53 | 1.84 |
| PLCG1 (Phospho-Tyr783) | *PLCG1* | P19174 | 1.81 | 1.00 | 1.82 |
| SMC1 (Phospho-Ser957) | *SMC1A* | Q14683 | 1.48 | 0.82 | 1.81 |
| Caspase 8 (Phospho-Ser347) | *CASP8* | Q14790 | 2.04 | 1.13 | 1.81 |
| HDAC2 (Phospho-Ser394) | *HDAC2* | Q92769 | 0.99 | 0.56 | 1.75 |
| Caspase 9 (Phospho-Thr125) | *CASP9* | P55211 | 0.87 | 0.50 | 1.73 |
| Smad3 (Phospho-Ser204) | *SMAD3* | P84022 | 0.81 | 0.48 | 1.70 |
| Ras-GRF1 (Phospho-Ser916) | *RASGRF1* | Q13972 | 0.71 | 0.43 | 1.66 |
| PKC zeta (Phospho-Thr560) | *PRKCZ* | Q05513 | 1.27 | 0.77 | 1.66 |
| ATF4 (Phospho-Ser245) | *ATF4* | P18848 | 4.26 | 2.65 | 1.60 |
| GAP43 (Phospho-Ser41) | *GAP43* | P17677 | 2.18 | 3.52 | 0.62 |
| GSK3 alpha (Phospho-Ser21) | *GSK3A* | P49840 | 0.69 | 1.13 | 0.61 |
| Caspase 6 (Phospho-Ser257) | *CASP6* | P55212 | 0.21 | 0.35 | 0.60 |
| Rho/Rac guanine nucleotide exchange factor 2 (Phospho-Ser885) | *ARHGEF2* | Q8TDA3 | 1.78 | 2.98 | 0.60 |
| NFkB-p100/p52 (Phospho-Ser869) | *NFKB2* | Q00653 | 0.45 | 0.75 | 0.59 |
| CDK2 (Phospho-Thr160) | *CDK2* | P24941 | 1.02 | 1.73 | 0.59 |
| PI3-kinase p85-subunit alpha/gamma (Phospho-Tyr467/Tyr199) | *PIK3R1/PIK3R3* | P27986/Q92569 | 3.23 | 5.47 | 0.59 |
| TYK2 (Phospho-Tyr1054) | *TYK2* | P29597 | 2.00 | 3.39 | 0.59 |
| IkB-beta (Phospho-Thr19) | *NFKBIB* | Q15653 | 1.99 | 3.37 | 0.59 |
| CD4 (Phospho-Ser433) | *CD4* | P01730 | 0.38 | 0.65 | 0.58 |
| MEK1 (Phospho-Ser221) | *MAP2K1* | Q02750 | 1.68 | 2.88 | 0.58 |
| AKT1 (Phospho-Ser246) | *AKT1* | P31749 | 0.77 | 1.32 | 0.58 |
| Shc (Phospho-Tyr427) | *SHC1* | P29353 | 0.31 | 0.53 | 0.58 |
| LAT (Phospho-Tyr171) | *LAT* | O43561 | 0.45 | 0.77 | 0.58 |
| BRCA1 (Phospho-Ser1524) | *BRCA1* | P38398 | 0.44 | 0.76 | 0.58 |
| Survivin (Phospho-Thr117) | *BIRC5* | O15392 | 1.07 | 1.87 | 0.57 |
| IL-10R-alpha (Phospho-Tyr496) | *IL10RA* | Q13651 | 1.71 | 2.99 | 0.57 |
| LKB1 (Phospho-Thr189) | *STK11* | Q15831 | 0.89 | 1.58 | 0.57 |
| ASK1 (Phospho-Ser966) | *MAP3K5* | Q99683 | 0.74 | 1.31 | 0.57 |
| CDC25A (Phospho-Ser75) | *CDC25A* | P30304 | 0.59 | 1.06 | 0.55 |
| CREB (Phospho-Ser133) | *CREB1* | P16220 | 1.35 | 2.48 | 0.54 |
| IKK-gamma (Phospho-Ser85) | *IKBKG* | Q9Y6K9 | 0.56 | 1.05 | 0.53 |
| Tau (Phospho-Thr212) | *MAPT* | P10636 | 0.36 | 0.68 | 0.53 |
| ACC1 (Phospho-Ser80) | *ACACA* | Q13085 | 0.08 | 0.15 | 0.53 |
| BID (Phospho-Ser78) | *BID* | P55957 | 0.36 | 0.68 | 0.53 |
| STAT6 (Phospho-Thr645) | *STAT6* | P42226 | 0.29 | 0.55 | 0.53 |
| MARCKS (Phospho-Ser163) | *MARCKS* | P29966 | 0.99 | 1.88 | 0.53 |
| VAV1 (Phospho-Tyr174) | *VAV1* | P15498 | 0.55 | 1.05 | 0.53 |
| ACC1 (Phospho-Ser79) | *ACACA* | Q13085 | 1.08 | 2.06 | 0.53 |
| P70S6K (Phospho-Thr229) | *RPS6KB1* | P23443 | 0.59 | 1.16 | 0.51 |
| ETK (Phospho-Tyr40) | *BMX* | P51813 | 0.62 | 1.26 | 0.50 |
| CDK7 (Phospho-Thr170) | *CDK7* | P50613 | 1.11 | 2.28 | 0.49 |
| PAK3 (Phospho-Ser154) | *PAK3* | O75914 | 0.75 | 1.54 | 0.49 |
| VASP (Phospho-Ser157) | *VASP* | P50552 | 0.74 | 1.54 | 0.48 |
| p27Kip1 (Phospho-Ser10) | *CDKN1B* | P46527 | 1.16 | 2.43 | 0.48 |
| eIF2A (Phospho-Ser51) | *EIF2S1* | P05198 | 0.62 | 1.31 | 0.47 |
| PKC alpha/beta II (Phospho-Thr638) | *PRKCA* | P17252 | 1.10 | 2.34 | 0.47 |
| Cortactin (Phospho-Tyr466) | *CTTN* | Q14247 | 0.69 | 1.47 | 0.47 |
| FAK (Phospho-Tyr861) | *PTK2* | Q05397 | 1.13 | 2.42 | 0.47 |
| PEA-15 (Phospho-Ser116) | *PEA15* | Q15121 | 0.94 | 2.09 | 0.45 |
| CDK1/CDC2 (Phospho-Thr14) | *CDK1* | P06493 | 1.17 | 2.64 | 0.44 |
| VEGFR2 (Phospho-Tyr1175) | *KDR* | P35968 | 0.51 | 1.16 | 0.44 |
| Raf1 (Phospho-Tyr341) | *RAF1* | P04049 | 0.53 | 1.21 | 0.44 |
| FKHR (Phospho-Ser256) | *FOXO1* | Q12778 | 0.98 | 2.24 | 0.44 |
| EGFR (Phospho-Tyr1016) | *EGFR* | P00533 | 0.82 | 2.00 | 0.41 |
| Smad2 (Phospho-Ser467) | *SMAD2* | Q15796 | 0.68 | 1.79 | 0.38 |
| Fos (Phospho-Thr232) | *FOS* | P01100 | 0.40 | 1.07 | 0.38 |
| c-Jun (Phospho-Thr93) | *JUN* | P05412 | 1.07 | 2.95 | 0.36 |
| Smad2 (Phospho-Thr220) | *SMAD2* | Q15796 | 1.21 | 3.70 | 0.33 |
| Raf1 (Phospho-Ser296) | *RAF1* | P04049 | 0.45 | 1.75 | 0.26 |
| Abl1 (Phospho-Thr754/735) | *ABL1* | P00519 | 0.58 | 2.67 | 0.22 |
| SLP-76 (Phospho-Tyr128) | *LCP2* | Q13094 | 0.35 | 1.76 | 0.20 |
| LYN (Phospho-Tyr507) | *LYN* | P07948 | 0.32 | 0.05 | 6.03 |
| c-Jun (Phospho-Tyr170) | *JUN* | P05412 | 2.90 | 0.72 | 4.04 |
| VASP (Phospho-Ser238) | *VASP* | P50552 | 3.62 | 1.14 | 3.18 |
| ERK3 (Phospho-Ser189) | *MAPK6* | Q16659 | 2.70 | 1.10 | 2.46 |
| ALK (Phospho-Tyr1604) | *ALK* | Q9UM73 | 4.97 | 2.04 | 2.43 |
| Kv1.3/KCNA3 (Phospho-Tyr135) | *KCNA3* | P22001 | 2.92 | 1.21 | 2.42 |
| Estrogen Receptor-alpha (Phospho-Ser167) | *ESR1* | P03372 | 4.17 | 1.73 | 2.41 |
| Myc (Phospho-Ser62) | *MYC* | P01106 | 2.68 | 1.13 | 2.37 |
| WASP (Phospho-Tyr290) | *WAS* | P42768 | 1.01 | 0.43 | 2.36 |
| AKT1 (Phospho-Tyr326) | *AKT1* | P31749 | 1.70 | 0.73 | 2.35 |
| Smad1 (Phospho-Ser465) | *SMAD1* | Q15797 | 0.99 | 0.43 | 2.29 |
| FKHRL1/FOXO3A (Phospho-Ser253) | *FOXO3* | O43524 | 1.66 | 0.73 | 2.28 |
| Keratin 18 (Phospho-Ser52) | *KRT18* | P05783 | 2.92 | 1.32 | 2.22 |
| Epo-R (Phospho-Tyr368) | *EPOR* | P19235 | 1.48 | 0.72 | 2.06 |
| Tau (Phospho-Ser396) | *MAPT* | P10636 | 1.07 | 0.52 | 2.05 |
| IL-13R/CD213a1 (Phospho-Tyr405) | *IL13RA1* | P78552 | 9.45 | 4.67 | 2.02 |
| KIT (Phospho-Tyr936) | *KIT* | P10721 | 3.67 | 1.83 | 2.01 |
| c-Jun (Phospho-Ser243) | *JUN* | P05412 | 2.01 | 1.02 | 1.96 |
| Rb (Phospho-Ser795) | *RB1* | P06400 | 3.22 | 1.69 | 1.91 |
| HSP90B (Phospho-Ser254) | *HSP90AB1* | P08238 | 0.96 | 0.52 | 1.86 |
| SP1 (Phospho-Thr739) | *SP1* | P08047 | 2.24 | 1.28 | 1.75 |
| IRS-1 (Phospho-Ser636) | *IRS1* | P35568 | 2.75 | 1.62 | 1.70 |
| EGFR (Phospho-Thr678) | *EGFR* | P00533 | 1.68 | 0.99 | 1.69 |
| Cyclin E1 (Phospho-Thr77) | *CCNE1* | P24864 | 0.13 | 0.08 | 1.63 |
| Raf1 (Phospho-Ser43) | *RAF1* | P04049 | 0.73 | 1.18 | 0.62 |
| p53 (Phospho-Ser378) | *TP53* | P04637 | 2.46 | 4.33 | 0.57 |
| CREB (Phospho-Ser142) | *CREB1* | P16220 | 1.14 | 2.00 | 0.57 |
| HER2 (Phospho-Tyr1221/Tyr1222) | *ERBB2* | P04626 | 0.42 | 0.73 | 0.57 |
| MKP-1 (Phospho-Ser359) | *DUSP1* | P28562 | 0.80 | 1.51 | 0.53 |
| FAK (Phospho-Tyr407) | *PTK2* | Q05397 | 1.00 | 1.95 | 0.51 |
| EGFR (Phospho-Thr693) | *EGFR* | P00533 | 0.49 | 0.98 | 0.50 |
| Src (Phospho-Ser75) | *SRC* | P12931 | 0.65 | 1.35 | 0.48 |
| CaMK4 (Phospho-Thr196/200) | *CAMK4* | Q16566 | 1.08 | 2.45 | 0.44 |
| CrkII (Phospho-Tyr221) | *CRK* | P46108 | 0.45 | 1.03 | 0.44 |
| PP2A-alpha (Phospho-Tyr307) | *PPP2CA* | P67775 | 0.38 | 0.90 | 0.42 |
| NFkB-p65 (Phospho-Ser468) | *RELA* | Q04206 | 0.89 | 2.17 | 0.41 |
| RSK1/2/3/4 (Phospho-Ser221/227/218/232) | *RPS6KA1/RPS6KA3/RPS6KA2* | Q15418/P51812/Q15349/Q9UK32 | 0.51 | 1.35 | 0.38 |
| Cyclin D3 (Phospho-Thr283) | *CCND3* | P30281 | 0.69 | 2.36 | 0.29 |
| Ezrin (Phospho-Tyr478) | *EZR* | P15311 | 1.05 | 3.75 | 0.28 |

HSF, human skin fibroblasts.

**Supplementary Table S3.** **Comparison of protein content in HaCat under micro-negative pressure and normal pressure**

| **Name** | **Gene symbol** | **Uniprot** | **HaCat negative pressure_Phos/ Unphos** | **HaCat normal pressure_Phos/ Unphos** | **FC ≥ 1.6_HaCat negative pressure _vs_HaCat normal pressure** |
| --- | --- | --- | --- | --- | --- |
| BRCA1 (Phospho-Ser1457) | *BRCA1* | P38398 | 2.80 | 0.31 | 9.04 |
| HDAC1 (Phospho-Ser421) | *HDAC1* | Q13547 | 0.84 | 0.13 | 6.43 |
| LYN (Phospho-Tyr507) | *LYN* | P07948 | 0.18 | 0.04 | 4.03 |
| GAP43 (Phospho-Ser41) | *GAP43* | P17677 | 3.46 | 1.09 | 3.18 |
| Cyclin B1 (Phospho-Ser147) | *CCNB1* | P14635 | 1.90 | 0.60 | 3.15 |
| Pyk2 (Phospho-Tyr881) | *PTK2B* | Q14289 | 1.52 | 0.54 | 2.82 |
| SYK (Phospho-Tyr348) | *SYK* | P43405 | 1.18 | 0.44 | 2.68 |
| CDC25C (Phospho-Ser216) | *CDC25C* | P30307 | 0.49 | 0.19 | 2.55 |
| IRS-1 (Phospho-Ser636) | *IRS1* | P35568 | 3.35 | 1.35 | 2.48 |
| S6 Ribosomal Protein (Phospho-Ser235) | *RPS6* | P62753 | 1.83 | 0.77 | 2.36 |
| Integrin beta-3 (Phospho-Tyr773) | *ITGB3* | P05106 | 2.29 | 1.00 | 2.28 |
| PP1 alpha (Phospho-Thr320) | *PPP1CA* | P62136 | 0.99 | 0.44 | 2.23 |
| PKD1/PKC mu (Phospho-Ser910) | *PRKD1* | Q15139 | 2.28 | 1.05 | 2.18 |
| Smad2 (Phospho-Ser467) | *SMAD2* | Q15796 | 1.20 | 0.55 | 2.17 |
| CK1-A (Phospho-Thr321) | *CSNK1A1* | P48729 | 5.06 | 2.34 | 2.17 |
| Tau (Phospho-Ser396) | *MAPT* | P10636 | 1.45 | 0.68 | 2.13 |
| Rb (Phospho-Ser795) | *RB1* | P06400 | 3.43 | 1.61 | 2.13 |
| ATF4 (Phospho-Ser245) | *ATF4* | P18848 | 3.80 | 1.81 | 2.10 |
| LKB1 (Phospho-Thr189) | *STK11* | Q15831 | 1.31 | 0.63 | 2.06 |
| BCL-2 (Phospho-Ser70) | *BCL2* | P10415 | 3.43 | 1.70 | 2.01 |
| GSK3 alpha/beta (Phospho-Tyr216/279) | *GSK3A/GSK3B* | P49840/P49841 | 2.43 | 1.22 | 1.98 |
| Caspase 9 (Phospho-Thr125) | *CASP9* | P55211 | 0.84 | 0.42 | 1.97 |
| ATP-Citrate Lyase (Phospho-Ser454) | *ACLY* | P53396 | 1.70 | 0.87 | 1.97 |
| EGFR (Phospho-Tyr869) | *EGFR* | P00533 | 0.69 | 0.35 | 1.95 |
| PKC delta (Phospho-Thr505) | *PRKCD* | Q05655 | 1.66 | 0.87 | 1.91 |
| Raf1 (Phospho-Ser43) | *RAF1* | P04049 | 1.69 | 0.88 | 1.91 |
| VASP (Phospho-Ser157) | *VASP* | P50552 | 2.12 | 1.12 | 1.89 |
| Met (Phospho-Tyr1234) | *MET* | P08581 | 2.52 | 1.35 | 1.87 |
| EGFR (Phospho-Thr693) | *EGFR* | P00533 | 1.27 | 0.68 | 1.86 |
| p44/42 MAPK (Phospho-Thr202) | *MAPK3* | P27361 | 1.81 | 1.00 | 1.81 |
| HSP90B (Phospho-Ser226) | *HSP90AB1* | P08238 | 7.96 | 4.43 | 1.79 |
| Pyk2 (Phospho-Tyr402) | *PTK2B* | Q14289 | 2.09 | 1.18 | 1.77 |
| Gab1 (Phospho-Tyr627) | *GAB1* | Q13480 | 3.02 | 1.71 | 1.77 |
| 4E-BP1 (Phospho-Thr45) | *EIF4EBP1* | Q13541 | 1.27 | 0.72 | 1.75 |
| p38 MAPK (Phospho-Tyr322) | *MAPK14* | Q16539 | 1.33 | 0.76 | 1.74 |
| P70S6K (Phospho-Thr421) | *RPS6KB1* | P23443 | 1.54 | 0.89 | 1.73 |
| Synaptotagmin (Phospho-Ser309) | *SYT1* | P21579 | 0.83 | 0.49 | 1.69 |
| BCL-2 (Phospho-Thr56) | *BCL2* | P10415 | 2.58 | 1.53 | 1.68 |
| STAM2 (Phospho-Tyr192) | *STAM2* | O75886 | 7.17 | 4.26 | 1.68 |
| Catenin beta (Phospho-Ser33) | *CTNNB1* | P35222 | 2.86 | 1.71 | 1.67 |
| Tau (Phospho-Thr231) | *MAPT* | P10636 | 2.85 | 1.76 | 1.62 |
| VEGFR2 (Phospho-Tyr1059) | *KDR* | P35968 | 1.72 | 1.06 | 1.62 |
| WASP (Phospho-Tyr290) | *WAS* | P42768 | 1.24 | 0.77 | 1.61 |
| ALK (Phospho-Tyr1507) | *ALK* | Q9UM73 | 4.09 | 2.55 | 1.61 |
| JAK1 (Phospho-Tyr1022) | *JAK1* | P23458 | 0.42 | 0.68 | 0.62 |
| Src (Phospho-Ser75) | *SRC* | P12931 | 0.61 | 0.97 | 0.62 |
| ATRIP (Phospho-Ser68/72) | *ATRIP* | Q8WXE1 | 0.12 | 0.19 | 0.62 |
| VEGFR2 (Phospho-Tyr1175) | *KDR* | P35968 | 0.51 | 0.83 | 0.62 |
| 4E-BP1 (Phospho-Ser65) | *EIF4EBP1* | Q13541 | 0.17 | 0.28 | 0.61 |
| STAT1 (Phospho-Ser727) | *STAT1* | P42224 | 0.80 | 1.31 | 0.61 |
| Smad3 (Phospho-Ser204) | *SMAD3* | P84022 | 0.36 | 0.60 | 0.61 |
| eNOS (Phospho-Ser1177) | *NOS3* | P29474 | 0.46 | 0.77 | 0.60 |
| KSR (Phospho-Ser392) | *KSR1* | Q8IVT5 | 0.89 | 1.50 | 0.60 |
| EPB41 (Phospho-Tyr418/660) | *EPB41* | P11171 | 0.70 | 1.17 | 0.60 |
| PAK1/2/3 (Phospho-Ser141) | *PAK1/2/3* | Q13153/Q13177/O75914 | 0.91 | 1.53 | 0.60 |
| Rel (Phospho-Ser503) | *REL* | Q04864 | 1.52 | 2.56 | 0.59 |
| Cyclin D3 (Phospho-Thr283) | *CCND3* | P30281 | 0.44 | 0.74 | 0.59 |
| HRS (Phospho-Tyr334) | *HGS* | O14964 | 1.05 | 1.78 | 0.59 |
| AurB (Phospho-Thr232) | *AURKB* | Q96GD4 | 0.99 | 1.68 | 0.59 |
| EGFR (Phospho-Ser1070) | *EGFR* | P00533 | 0.51 | 0.87 | 0.58 |
| Stathmin 1 (Phospho-Ser15) | *STMN1* | P16949 | 0.84 | 1.44 | 0.58 |
| CDK2 (Phospho-Thr160) | *CDK2* | P24941 | 0.78 | 1.34 | 0.58 |
| P70S6K (Phospho-Ser371) | *RPS6KB1* | P23443 | 0.95 | 1.67 | 0.57 |
| GRK1 (Phospho-Ser21) | *GRK1* | Q15835 | 1.13 | 2.00 | 0.57 |
| MARCKS (Phospho-Ser163) | *MARCKS* | P29966 | 0.79 | 1.41 | 0.56 |
| DAPP1 (Phospho-Tyr139) | *DAPP1* | Q9UHF2 | 0.66 | 1.17 | 0.56 |
| EGFR (Phospho-Tyr1172) | *EGFR* | P00533 | 0.15 | 0.27 | 0.56 |
| Estrogen Receptor-alpha (Phospho-Ser104) | *ESR1* | P03372 | 0.93 | 1.66 | 0.56 |
| PKC pan activation site (Phospho) | *PRKCA/PRKCB/PRKCD/*  *PRKCE/PRKCG/PRKCH****/***  *PRKCQ/PRKCZ* | P17252/P05771/Q05655/  Q02156/P05129/P24723/  Q04759/Q05513 | 0.76 | 1.36 | 0.56 |
| SLP-76 (Phospho-Tyr128) | *LCP2* | Q13094 | 0.53 | 0.96 | 0.55 |
| PTEN (Phospho-Ser380) | *PTEN* | P60484 | 0.40 | 0.74 | 0.54 |
| GATA1 (Phospho-Ser142) | *GATA1* | P15976 | 0.70 | 1.31 | 0.54 |
| HSP27 (Phospho-Ser15) | *HSPB1* | P04792 | 0.89 | 1.66 | 0.54 |
| p130Cas (Phospho-Tyr410) | *BCAR1* | P56945 | 0.60 | 1.12 | 0.53 |
| BRCA1 (Phospho-Ser1524) | *BRCA1* | P38398 | 0.48 | 0.90 | 0.53 |
| Rb (Phospho-Ser780) | *RB1* | P06400 | 0.26 | 0.50 | 0.53 |
| c-Jun (Phospho-Ser63) | *JUN* | P05412 | 0.61 | 1.16 | 0.53 |
| VAV1 (Phospho-Tyr174) | *VAV1* | P15498 | 0.58 | 1.11 | 0.52 |
| GluR1 (Phospho-Ser863) | *GRIA1* | P42261 | 1.18 | 2.29 | 0.52 |
| Raf1 (Phospho-Ser259) | *RAF1* | P04049 | 0.88 | 1.71 | 0.52 |
| p53 (Phospho-Ser6) | *TP53* | P04637 | 1.03 | 1.99 | 0.51 |
| HDAC3 (Phospho-Ser424) | *HDAC3* | O15379 | 0.26 | 0.50 | 0.51 |
| NMDAR2B (Phospho-Tyr1472) | *GRIN2B* | Q13224 | 0.13 | 0.25 | 0.51 |
| Tau (Phospho-Ser235) | *MAPT* | P10636 | 0.08 | 0.15 | 0.51 |
| ATPase (Phospho-Ser16) | *ATP1A1* | P05023 | 0.93 | 1.87 | 0.50 |
| Paxillin (Phospho-Tyr31) | *PXN* | P49023 | 0.75 | 1.51 | 0.50 |
| CDK1/CDC2 (Phospho-Thr14) | *CDK1* | P06493 | 1.17 | 2.37 | 0.49 |
| AKT1 (Phospho-Ser124) | *AKT1* | P31749 | 0.46 | 0.93 | 0.49 |
| MKK6/MAP2K6 (Phospho-Ser207) | *MAP2K6* | P52564 | 0.32 | 0.65 | 0.49 |
| p53 (Phospho-Ser315) | *TP53* | P04637 | 0.30 | 0.62 | 0.48 |
| 14-3-3 zeta (Phospho-Ser58) | *YWHAZ* | P63104 | 1.09 | 2.26 | 0.48 |
| CDC25A (Phospho-Ser75) | *CDC25A* | P30304 | 0.59 | 1.22 | 0.48 |
| PPAR-BP (Phospho-Thr1457) | *MED1* | Q15648 | 0.46 | 0.96 | 0.48 |
| FGFR1 (Phospho-Tyr766) | *FGFR1* | P11362 | 0.46 | 0.97 | 0.48 |
| Epo-R (Phospho-Tyr368) | *EPOR* | P19235 | 0.80 | 1.68 | 0.48 |
| IRS-1 (Phospho-Ser312) | *IRS1* | P35568 | 0.64 | 1.35 | 0.48 |
| p53 (Phospho-Thr18) | *TP53* | P04637 | 1.34 | 2.80 | 0.48 |
| JAK2 (Phospho-Tyr221) | *JAK2* | O60674 | 0.49 | 1.04 | 0.47 |
| MEK1 (Phospho-Ser217) | *MAP2K1* | Q02750 | 0.51 | 1.10 | 0.47 |
| Abl1 (Phospho-Tyr204) | *ABL1* | P00519 | 0.09 | 0.19 | 0.45 |
| HSL (Phospho-Ser554) | *LIPE* | Q05469 | 0.41 | 0.91 | 0.45 |
| Smad3 (Phospho-Ser213) | *SMAD3* | P84022 | 2.05 | 4.71 | 0.44 |
| B-RAF (Phospho-Ser446) | *BRAF* | P15056 | 1.02 | 2.39 | 0.43 |
| Ezrin (Phospho-Thr566) | *EZR* | P15311 | 0.17 | 0.41 | 0.41 |
| Cortactin (Phospho-Tyr421) | *CTTN* | Q14247 | 1.36 | 3.38 | 0.40 |
| P70S6K (Phospho-Ser418) | *RPS6KB1* | P23443 | 1.11 | 2.78 | 0.40 |
| VEGFR2 (Phospho-Tyr1214) | *KDR* | P35968 | 0.06 | 0.16 | 0.39 |
| IKK-gamma (Phospho-Ser31) | *IKBKG* | Q9Y6K9 | 0.23 | 0.61 | 0.38 |
| STAT3 (Phospho-Ser727) | *STAT3* | P40763 | 1.04 | 2.77 | 0.38 |
| PDGFR alpha (Phospho-Tyr849) | *PDGFRA* | P16234 | 1.10 | 2.98 | 0.37 |
| ERK3 (Phospho-Ser189) | *MAPK6* | Q16659 | 1.24 | 3.42 | 0.36 |
| HNF4 alpha (Phospho-Ser313) | *HNF4A* | P41235 | 0.69 | 1.91 | 0.36 |
| ASK1 (Phospho-Ser83) | *MAP3K5* | Q99683 | 1.11 | 3.09 | 0.36 |
| DNA-PK (Phospho-Thr2647) | *PRKDC* | P78527 | 0.69 | 1.93 | 0.35 |
| PKC delta (Phospho-Ser645) | *PRKCD* | Q05655 | 0.42 | 1.20 | 0.35 |
| Amyloid beta A4 (Phospho-Thr743/668) | *APP* | P05067 | 0.52 | 1.51 | 0.35 |
| BTK (Phospho-Tyr223) | *BTK* | Q06187 | 0.40 | 1.15 | 0.35 |
| CREB (Phospho-Ser142) | *CREB1* | P16220 | 0.55 | 1.68 | 0.33 |
| Chk2 (Phospho-Thr383) | *CHEK2* | O96017 | 0.34 | 1.05 | 0.32 |
| ACC1 (Phospho-Ser80) | *ACACA* | Q13085 | 0.17 | 0.57 | 0.30 |
| Synuclein alpha (Phospho-Tyr133) | *SNCA* | P37840 | 0.36 | 1.44 | 0.25 |
| RSK1/2/3/4 (Phospho-Ser221/227/218/232) | *RPS6KA1/RPS6KA3/*  *RPS6KA2* | Q15418/P51812/Q15349/  Q9UK32 | 0.28 | 1.26 | 0.22 |
| IKK-alpha/beta (Phospho-Ser180/181) | *CHUK/IKBKB* | O15111/O14920 | 1.57 | 0.50 | 3.12 |
| NFkB-p65 (Phospho-Thr254) | *RELA* | Q04206 | 2.49 | 0.80 | 3.11 |
| AFX/FOXO4 (Phospho-Ser197) | *FOXO4* | P98177 | 1.52 | 0.56 | 2.72 |
| HDAC5 (Phospho-Ser259) | *HDAC5* | Q9UQL6 | 1.34 | 0.53 | 2.55 |
| PKR (Phospho-Thr451) | *EIF2AK2* | P19525 | 2.01 | 0.90 | 2.23 |
| BAD (Phospho-Ser136) | *BAD* | Q92934 | 1.60 | 0.78 | 2.06 |
| LKB1 (Phospho-Ser428) | *STK11* | Q15831 | 1.89 | 0.92 | 2.05 |
| Tyrosine Hydroxylase (Phospho-Ser40) | *TH* | P07101 | 1.33 | 0.65 | 2.05 |
| Estrogen Receptor-alpha (Phospho-Ser167) | *ESR1* | P03372 | 2.96 | 1.45 | 2.04 |
| AKT1 (Phospho-Tyr326) | *AKT1* | P31749 | 1.32 | 0.66 | 1.99 |
| ACC1 (Phospho-Ser79) | *ACACA* | Q13085 | 2.28 | 1.17 | 1.95 |
| 4E-BP1 (Phospho-Thr70) | *EIF4EBP1* | Q13541 | 0.87 | 0.45 | 1.91 |
| PDGFR beta (Phospho-Tyr751) | *PDGFRB* | P09619 | 1.73 | 0.91 | 1.91 |
| PKC epsilon (Phospho-Ser729) | *PRKCE* | Q02156 | 1.46 | 0.78 | 1.88 |
| CDK7 (Phospho-Thr170) | *CDK7* | P50613 | 1.72 | 0.96 | 1.78 |
| MKK7/MAP2K7 (Phospho-Ser271) | *MAP2K7* | O14733 | 1.22 | 0.70 | 1.74 |
| Ret (Phospho-Tyr905) | *RET* | P07949 | 2.66 | 1.54 | 1.73 |
| MEK1 (Phospho-Ser298) | *MAP2K1* | Q02750 | 0.70 | 0.41 | 1.73 |
| PAK1/2 (Phospho-Ser199) | *PAK1/2* | Q13153/Q13177 | 0.93 | 0.54 | 1.72 |
| PPAR-gamma (Phospho-Ser112) | *PPARG* | P37231 | 0.12 | 0.07 | 1.72 |
| DAB1 (Phospho-Tyr220) | *DAB1* | O75553 | 2.56 | 1.51 | 1.70 |
| p53 (Phospho-Ser378) | *TP53* | P04637 | 2.27 | 1.35 | 1.69 |
| Myosin regulatory light chain 2 (Phospho-Ser18) | *MYL2* | P10916 | 1.22 | 0.73 | 1.68 |
| Calmodulin (Phospho-Thr79/Ser81) | *CALM1* | P62158 | 1.15 | 0.70 | 1.65 |
| Tau (Phospho-Ser356) | *MAPT* | P10636 | 0.16 | 0.10 | 1.63 |
| Integrin beta-4 (Phospho-Tyr1510) | *ITGB4* | P16444 | 1.74 | 1.07 | 1.62 |
| HDAC5 (Phospho-Ser498) | *HDAC5* | Q9UQL6 | 1.07 | 1.75 | 0.61 |
| FKHRL1/FOXO3A (Phospho-Ser253) | *FOXO3* | O43524 | 0.56 | 0.96 | 0.58 |
| Raf1 (Phospho-Ser338) | *RAF1* | P04049 | 0.32 | 0.56 | 0.57 |
| Fos (Phospho-Thr232) | *FOS* | P01100 | 0.39 | 0.88 | 0.45 |
| BAD (Phospho-Ser91/128) | *BAD* | Q92934 | 0.54 | 2.28 | 0.24 |

Notes:

| Name | Locus name |
| --- | --- |
| Gene symbol | Locus protein gene symbol |
| Uniprot | Locus protein Uniprot No. |
| Sample Name_Phospho/Unphospho | Phosphorylation level of each antibody in the group = the mean signal value of the phosphorylation locus of the antibody/the mean signal value of the corresponding non-phosphorylation locus |
| MNPDD Group_vs_CG_Ratio | Ratio of the phosphorylation level of the same phosphorylation locus protein between the two groups |
| FC≥X_ MNPDD Group vs CG | In the MNPDD group vs. the control group, the phosphorylation locus protein obtained by setting the ratio between groups ≥ x or ≤ 1 / X (X represents the modulation multiple between groups. When X was set, the number of phosphorylation locus protein of the modulation difference between groups was controlled at approximately 10–30% of the total number of detected proteins) |
| Red color refers to increasing by more than X times; green color refers to reducing by more than X times; orange color refers to a locus protein with high dispersion of the signal value of two repeats between groups. The ratio result was only used for reference.  CG, control group; HaCat, human immortalized keratinocyte cells; MNPDD, micro-negative pressure drainage dressing. | |

**Supplementary Table S4. Analysis of HSF cell micro-negative pressure vs. HSF cell normal pressure KEGG pathways**

| **Category** | **Term** | **Count** | **Genes** | ***P* value** | **Fold enrichment** |
| --- | --- | --- | --- | --- | --- |
| KEGG_PATHWAY | hsa04151:PI3K-Akt signaling pathway | 35 | *HSP90AB1, STK11, RPS6KB1, FOXO3, KIT, PTEN, AKT1, CCNE1, PTK2, CASP9, PPP2CA, PIK3R3, MYC, CHUK, PIK3R1, PRKCA, EGFR, MAP2K1, CREB1, RELA, TP53, RAF1, BAD, IRS1, BRCA1, CDK2, KDR, ATF4, CDKN1B, CD19, CCND3, IKBKG, PDGFRA, EPOR, IKBKB* | 0.00 | 6.46 |
| KEGG_PATHWAY | hsa04010:MAPK signaling pathway | 28 | *NFKB2, AKT1, FOS, MAP3K5, PAK2, MAPT, MYC, CHUK, PRKCA, EGFR, MAP2K1, RELA, MAP2K3, TP53, RAF1, ATF4, RPS6KA3, DUSP1, RPS6KA1, RASGRF1, RPS6KA2, MAPK14, JUN, IKBKG, PDGFRA, STMN1, IKBKB, CRK* | 0.00 | 7.05 |
| KEGG_PATHWAY | hsa04722:Neurotrophin signaling pathway | 24 | *MAP2K1, NFKBIB, RELA, TP53, RAF1, BAD, FOXO3, IRS1, AKT1, MAP3K5, RPS6KA3, ATF4, CAMK4, PLCG1, RPS6KA1, RPS6KA2, JUN, MAPK14, SHC1, IKBKB, ABL1, PIK3R3, CRK, PIK3R1* | 0.00 | 12.74 |
| KEGG_PATHWAY | hsa04510:Focal adhesion | 24 | *PRKCA, EGFR, MAP2K1, BCAR1, ERBB2, RAF1, BAD, VAV1, PTEN, VASP, SRC, KDR, AKT1, PTK2, CCND3, PAK2, RASGRF1, PAK3, JUN, PDGFRA, SHC1, PIK3R3, CRK, PIK3R1* | 0.00 | 7.42 |
| KEGG_PATHWAY | hsa04062:Chemokine signaling pathway | 23 | *PRKCZ, MAP2K1, LYN, NFKBIB, BCAR1, RELA, RAF1, FOXO3, WAS, VAV1, SRC, AKT1, PTK2, PLCB3, GSK3A, PTK2B, IKBKG, SHC1, IKBKB, PIK3R3, CRK, CHUK, PIK3R1* | 0.00 | 7.88 |
| KEGG_PATHWAY | hsa04012:ErbB signaling pathway | 21 | *PRKCA, EGFR, MAP2K1, ERBB2, RAF1, RPS6KB1, BAD, SRC, AKT1, PTK2, CDKN1B, PAK2, PLCG1, PAK3, JUN, SHC1, ABL1, PIK3R3, MYC, CRK, PIK3R1* | 0.00 | 15.37 |
| KEGG_PATHWAY | hsa04660:T cell receptor signaling pathway | 21 | *MAP2K1, NFKBIB, RELA, CD247, RAF1, VAV1, AKT1, LAT, FOS, PAK2, PLCG1, PAK3, JUN, MAPK14, IKBKG, CD4, IKBKB, PIK3R3, CHUK, PIK3R1, LCP2* | 0.00 | 13.38 |
| KEGG_PATHWAY | hsa04110:Cell cycle | 17 | *CDK1, TP53, SMAD3, CHEK1, SMAD2, CDK7, RB1, CDC25A, CDK2, CCNB1, CCNE1, CDKN1B, HDAC2, CCND3, SMC1A, ABL1, MYC* | 0.00 | 8.73 |
| KEGG_PATHWAY | hsa04810:Regulation of actin cytoskeleton | 15 | *EGFR, MAP2K1, BCAR1, RAF1, VAV1, WAS, SRC, PTK2, EZR, PAK2, PAK3, PDGFRA, PIK3R3, CRK, PIK3R1* | 0.00 | 4.55 |
| KEGG_PATHWAY | hsa04931:Insulin resistance | 14 | *PRKCZ, CREB1, RELA, FOXO1, RPS6KB1, IRS1, PTEN, AKT1, RPS6KA3, RPS6KA1, RPS6KA2, IKBKB, PIK3R3, PIK3R1* | 0.00 | 8.26 |
| KEGG_PATHWAY | hsa04910:Insulin signaling pathway | 14 | *AKT1, PRKCZ, MAP2K1, ACACA, RAF1, FOXO1, SHC1, RPS6KB1, BAD, IKBKB, PIK3R3, CRK, IRS1, PIK3R1* | 0.00 | 6.46 |
| KEGG_PATHWAY | hsa04370:VEGF signaling pathway | 13 | *PRKCA, AKT1, PTK2, CASP9, PLCG1, MAP2K1, MAPK14, RAF1, BAD, PIK3R3, PIK3R1, SRC, KDR* | 0.00 | 13.57 |
| KEGG_PATHWAY | hsa04210:Apoptosis | 13 | *BID, RELA, TP53, BAD, AKT1, CASP6, CASP9, IKBKG, CASP8, PIK3R3, IKBKB, PIK3R1, CHUK* | 0.00 | 13.36 |
| KEGG_PATHWAY | hsa04150:mTOR signaling pathway | 12 | *PRKCA, AKT1, RPS6KA3, RPS6KA1, RPS6KA2, STK11, RPS6KB1, IKBKB, PIK3R3, PTEN, IRS1, PIK3R1* | 0.00 | 13.18 |
| KEGG_PATHWAY | hsa04115:p53 signaling pathway | 11 | *BID, CCNB1, CCNE1, CDK1, CCND3, CASP9, CASP8, TP53, CHEK1, PTEN, CDK2* | 0.00 | 10.46 |
| KEGG_PATHWAY | hsa04152:AMPK signaling pathway | 11 | *AKT1, STK11, PPP2CA, CREB1, ACACA, FOXO1, RPS6KB1, FOXO3, PIK3R3, IRS1, PIK3R1* | 0.00 | 5.70 |
| KEGG_PATHWAY | hsa04630:Jak-STAT signaling pathway | 10 | *STAT6, AKT1, TYK2, CCND3, IL10RA, EPOR, PIK3R3, IL13RA1, MYC, PIK3R1* | 0.00 | 4.39 |
| KEGG_PATHWAY | hsa04020:Calcium signaling pathway | 9 | *PRKCA, EGFR, PLCB3, CAMK4, PLCG1, PTK2B, ERBB2, PDGFRA, RYR2* | 0.01 | 3.20 |
| KEGG_PATHWAY | hsa04920:Adipocytokine signaling pathway | 8 | *AKT1, STK11, RELA, NFKBIB, IKBKG, IKBKB, IRS1, CHUK* | 0.00 | 7.28 |
| KEGG_PATHWAY | hsa04350:TGF-beta signaling pathway | 7 | *SP1, PPP2CA, SMAD3, SMAD2, RPS6KB1, SMAD1, MYC* | 0.00 | 5.31 |
| KEGG_PATHWAY | hsa04911:Insulin secretion | 5 | *PRKCA, ATF4, PLCB3, CREB1, RYR2* | 0.04 | 3.75 |

HSF, human skin fibroblasts; KEGG, Kyoto Encyclopedia of Genes and Genomes.

**Supplementary Table S5. Analysis of HaCat cell micro-negative pressure vs. HaCat cell normal pressure KEGG pathways**

| **Category** | **Term** | **Count** | **Genes** | ***P* value** | **Fold enrichment** |
| --- | --- | --- | --- | --- | --- |
| KEGG_PATHWAY | hsa04151:PI3K-Akt signaling pathway | 41 | *HSP90AB1, FGFR1, YWHAZ, STK11, ITGB4, RPS6KB1, FOXO3, ITGB3, PTEN, AKT1, EIF4EBP1, CASP9, BCL2, NOS3, CHUK, SYK, PRKCA, EGFR, MAP2K1, CREB1, RELA, MET, TP53, RAF1, BAD, RPS6, IRS1, CDK2, BRCA1, KDR, ATF4, CCND3, GSK3B, MAPK3, IKBKG, PDGFRA, JAK1, PDGFRB, JAK2, EPOR, IKBKB* | 0.00 | 6.65 |
| KEGG_PATHWAY | hsa04510:Focal adhesion | 32 | *MYL2, BCAR1, ITGB4, ITGB3, PTEN, PXN, SRC, CTNNB1, AKT1, PAK2, PAK3, BCL2, PAK1, PRKCA, EGFR, MAP2K1, BRAF, MET, RAF1, PRKCG, BAD, VAV1, VASP, KDR, PRKCB, PPP1CA, CCND3, JUN, GSK3B, MAPK3, PDGFRA, PDGFRB* | 0.00 | 8.69 |
| KEGG_PATHWAY | hsa04010:MAPK signaling pathway | 29 | *FGFR1, AKT1, FOS, MAP3K5, PAK2, MAPT, PAK1, MAP2K7, MAP2K6, CHUK, PRKCA, EGFR, MAP2K1, BRAF, RELA, TP53, RAF1, PRKCG, PRKCB, ATF4, MAPK14, JUN, IKBKG, MAPK3, PDGFRA, PDGFRB, HSPB1, STMN1, IKBKB* | 0.00 | 6.41 |
| KEGG_PATHWAY | hsa04062:Chemokine signaling pathway | 26 | *PRKCZ, BCAR1, FOXO3, PXN, SRC, AKT1, PTK2B, PAK1, CHUK, MAP2K1, BRAF, LYN, RELA, RAF1, STAT1, PRKCD, WAS, VAV1, STAT3, GSK3A, GSK3B, IKBKG, MAPK3, JAK2, IKBKB, GRK1* | 0.00 | 7.82 |
| KEGG_PATHWAY | hsa04722:Neurotrophin signaling pathway | 22 | *BRAF, MAP2K1, RELA, TP53, RAF1, BAD, FOXO3, PRKCD, IRS1, AKT1, MAP3K5, ATF4, JUN, MAPK14, BCL2, GSK3B, GAB1, MAPK3, IKBKB, ABL1, MAP2K7, CALM1* | 0.00 | 10.25 |
| KEGG_PATHWAY | hsa04012:ErbB signaling pathway | 21 | *PRKCA, EGFR, BRAF, MAP2K1, RAF1, RPS6KB1, PRKCG, BAD, SRC, PRKCB, AKT1, EIF4EBP1, PAK2, PAK3, GSK3B, JUN, GAB1, MAPK3, PAK1, ABL1, MAP2K7* | 0.00 | 13.50 |
| KEGG_PATHWAY | hsa04810:Regulation of actin cytoskeleton | 21 | *EGFR, FGFR1, MYL2, MAP2K1, BRAF, BCAR1, ITGB4, RAF1, ITGB3, WAS, VAV1, PXN, SRC, PPP1CA, EZR, PAK2, PAK3, MAPK3, PDGFRA, PDGFRB, PAK1* | 0.00 | 5.59 |
| KEGG_PATHWAY | hsa04660:T cell receptor signaling pathway | 19 | *MAP2K1, RELA, RAF1, VAV1, AKT1, PRKCQ, FOS, PAK2, PAK3, JUN, MAPK14, GSK3B, MAPK3, IKBKG, PAK1, IKBKB, MAP2K7, CHUK, LCP2* | 0.00 | 10.63 |
| KEGG_PATHWAY | hsa04110:Cell cycle | 17 | *CDK1, YWHAZ, TP53, SMAD3, PRKDC, SMAD2, CDK7, CHEK2, RB1, CDC25C, CDC25A, CDK2, CCNB1, CCND3, HDAC1, GSK3B, ABL1* | 0.00 | 7.67 |
| KEGG_PATHWAY | hsa04910:Insulin signaling pathway | 17 | *PRKCZ, BRAF, MAP2K1, ACACA, RAF1, RPS6KB1, BAD, RPS6, IRS1, AKT1, PPP1CA, EIF4EBP1, GSK3B, MAPK3, IKBKB, LIPE, CALM1* | 0.00 | 6.89 |
| KEGG_PATHWAY | hsa04370:VEGF signaling pathway | 15 | *PRKCA, MAP2K1, RAF1, PRKCG, BAD, PXN, SRC, KDR, PRKCB, AKT1, CASP9, MAPK14, MAPK3, HSPB1, NOS3* | 0.00 | 13.75 |
| KEGG_PATHWAY | hsa04931:Insulin resistance | 15 | *PRKCZ, RELA, CREB1, RPS6KB1, PRKCE, PRKCD, IRS1, PTEN, STAT3, AKT1, PRKCQ, PPP1CA, GSK3B, NOS3, IKBKB* | 0.00 | 7.77 |
| KEGG_PATHWAY | hsa04150:mTOR signaling pathway | 13 | *PRKCA, AKT1, EIF4EBP1, BRAF, STK11, MAPK3, PRKCG, RPS6KB1, IKBKB, RPS6, PTEN, IRS1, PRKCB* | 0.00 | 12.54 |
| KEGG_PATHWAY | hsa04152:AMPK signaling pathway | 11 | *AKT1, EIF4EBP1, HNF4A, STK11, CREB1, PPARG, ACACA, RPS6KB1, FOXO3, IRS1, LIPE* | 0.00 | 5.00 |
| KEGG_PATHWAY | hsa04920:Adipocytokine signaling pathway | 10 | *AKT1, PRKCQ, STK11, RELA, IKBKG, JAK2, IKBKB, IRS1, CHUK, STAT3* | 0.00 | 7.99 |
| KEGG_PATHWAY | hsa04144:Endocytosis | 10 | *EGFR, PRKCZ, STAM2, PDGFRA, SMAD3, HGS, SMAD2, WAS, SRC, GRK1* | 0.03 | 2.32 |
| KEGG_PATHWAY | hsa04210:Apoptosis | 9 | *AKT1, CASP9, RELA, BCL2, IKBKG, TP53, BAD, IKBKB, CHUK* | 0.00 | 8.12 |
| KEGG_PATHWAY | hsa04310:Wnt signaling pathway | 9 | *CSNK1A1, PRKCA, CCND3, JUN, GSK3B, TP53, PRKCG, CTNNB1, PRKCB* | 0.00 | 3.65 |
| KEGG_PATHWAY | hsa04020:Calcium signaling pathway | 9 | *PRKCA, EGFR, PTK2B, PDGFRA, PDGFRB, PRKCG, NOS3, PRKCB, CALM1* | 0.01 | 2.81 |
| KEGG_PATHWAY | hsa04115:p53 signaling pathway | 8 | *CCNB1, CDK1, CCND3, CASP9, TP53, CHEK2, PTEN, CDK2* | 0.00 | 6.68 |
| KEGG_PATHWAY | hsa04630:Jak-STAT signaling pathway | 8 | *AKT1, CCND3, STAM2, JAK1, EPOR, JAK2, STAT1, STAT3* | 0.01 | 3.09 |
| KEGG_PATHWAY | hsa04911:Insulin secretion | 6 | *PRKCA, ATF4, CREB1, PRKCG, ATP1A1, PRKCB* | 0.02 | 3.95 |

HaCat, human immortalized keratinocyte cells; KEGG, Kyoto Encyclopedia of Genes and Genomes.
